# Study on inter-segment interference mechanisms and patterns between horizontal well sections in a combined well pattern of horizontal and vertical wells in offshore oilfields

**DOI:** 10.1038/s41598-026-41737-3

**Published:** 2026-03-02

**Authors:** Ma Kuiqian, Zhang Zhang, Wang Lilei, Deng Jingfu, Zhang Yunlai, Sun Qiang, Zhang Xuemin, Mingxing Sun, Dou Xiangji, Zhang Xiangkun, Wu Longzhi

**Affiliations:** 1 CNOOC China Ltd., Tianjin Branch, Tianjin, 300452 China; 2 CNOOC EnerTech-Drilling & Production Co., Tianjin, 300452 China; 3 Changzhou University, Changzhou, 213000 China

**Keywords:** composite well pattern, Interference between horizontal and vertical wells, Inter-segment interference, Main controlling factors, Remaining oil distribution, Engineering, Hydrology

## Abstract

In the development of heavy oil fields in the Bohai Sea, the “horizontal + directional” well pattern has significantly improved recovery rates. However, as the oilfield enters the ultra-high water cut stage, changes in waterflooding behavior and production characteristics lead to a more complex distribution of remaining oil, posing challenges for subsequent development. This study introduces a dynamic interference analysis method that integrates three-dimensional (3D) physical modeling with numerical simulation. The method optimizes the interference prediction model under the “horizontal + directional” well pattern, aiding in well placement optimization and enhancing development efficiency during the ultra-high water cut period. Additionally, a phenomenon was observed where remaining oil concentrates in the central segment during the displacement process. Through numerical simulations under varying permeability rhythms, the impact of the injection-production relationship on the distribution of remaining oil was revealed, providing a theoretical foundation for well pattern optimization. The findings offer technical support for the continued development of Bohai Oilfields under ultra-high water cut conditions and provide valuable guidance for other offshore heavy oil fields. The new methods proposed in this study can improve recovery rates in complex waterflood environments.

## Introduction

The Bohai Oilfield, as one of China’s most important offshore oilfields, has achieved significant development success over the past decades through continuous technological innovation. However, with the field entering the ultra-high water cut stage, fundamental shifts in development challenges have emerged. During this phase, the remaining oil exhibits a “highly dispersed overall but locally enriched” distribution pattern, waterflood efficiency declines sharply, and conventional adjustment measures are largely ineffective, severely limiting the potential for improving recovery^1^. Therefore, identifying the dominant factors controlling remaining oil distribution and developing effective exploitation strategies during the ultra-high water cut stage has become a critical issue in current field development^2^.

Under the combined well network development mode, particularly in complex configurations integrating horizontal and directional wells, interference between injection and production wells presents new characteristics. While this approach improves overall recovery, multi-segment interference often leads to uneven water front propagation, altering both the macroscopic distribution and microscopic occurrence of remaining oil. Previous experimental and numerical studies have highlighted the relationship between injection-production interference and waterflood efficiency. For example, three-dimensional large-scale physical model experiments have examined remaining oil and waterflood response^1^, and multi-point refracturing mechanism analyses have demonstrated significant impacts of interference on displacement performance. Quantitative characterization and recovery measures of horizontal-well in-layer interference have also been proposed^3–5^.

However, these studies primarily focus on local well-group responses or specific well patterns, lacking systematic investigation of inter-segment interference mechanisms at the scale of full well networks. Some studies have used numerical simulation to analyze intra-layer interference and sweep efficiency^6,7^, but these approaches are often based on idealized models or single-factor assumptions, failing to accurately capture the dynamic coupling of multiple factors in strongly heterogeneous reservoirs. Furthermore, under ultra-high water cut conditions, existing models seldom integrate three-dimensional physical experiments with actual production data, limiting quantitative understanding of interference intensity, remaining oil enrichment, and their relationship with well pattern parameters. Although some research has explored the effects of lithologic rhythm^8^ or permeability contrast^9,10^ on remaining oil distribution, scalable predictive models and operational strategies remain insufficient, restricting guidance for well-network optimization and exploitation planning.

Particularly under the “horizontal–directional” interference pattern formed between injection and production wells, the coupling of segmental permeability differences and injection-production pressure differentials often promotes preferential flow along low-resistance paths, intensifying ineffective circulation. This issue is especially pronounced in strongly heterogeneous reservoirs, where large permeability contrasts exacerbate the suppression of low-permeability layers by high-permeability layers, further complicating remaining oil distribution and significantly limiting ultimate recovery. Recent studies on CO_2_ pre-fracturing and multi-component gas migration in nanopores provide new insights into enhancing displacement efficiency and optimizing injection strategies, which could be extended to offshore heavy-oil reservoirs^11,12^.

To address the aforementioned limitations, this study focuses on a representative strongly heterogeneous reservoir in the Bohai Oilfield and innovatively integrates three-dimensional physical model experiments with comprehensive numerical simulations. After validating the reliability of the numerical model, multiple simulation scenarios were designed to elucidate the controlling influence of injection–production relationships on remaining oil distribution. Based on these results, an inter-segment interference index model considering multi-factor coupling was developed to quantitatively characterize interference intensity and remaining oil enrichment zones, providing a theoretical basis for well-network optimization and flow-field management. The findings offer robust technical support for accurate prediction and efficient exploitation of remaining oil during the ultra-high water cut stage in the Bohai Oilfield and provide valuable guidance for the development of other offshore heavy-oil reservoirs.

## Methodology

To investigate the impact of the horizontal-vertical interference pattern on remaining oil enrichment in the Bohai Oilfield during the ultra-high water cut period, this study employed an approach integrating physical and numerical simulations. The specific methodology is outlined below.

### Physical simulation

The physical simulation employs a three-dimensional physical model to replicate waterflooding processes in reservoirs and inter-segment interference within injection-production layers. Based on the actual reservoir characteristics of Bohai Oilfield’s QHD32-6 block, the model utilizes cores with varying permeability to simulate heterogeneous reservoirs, with a focus on investigating inter-segment interference effects on waterflooding. After constructing the physical simulation model, experimental crude oil and mineralized water were selected through analysis of Bohai Oilfield’s field production data. To ensure experimental rigor, two scenarios with different permeability gradients were designed for comparison, with the optimal configuration chosen as the control group. The final phase involved designing experimental procedures and establishing the experimental platform.

#### Model construction approach

**Fig. 1:**
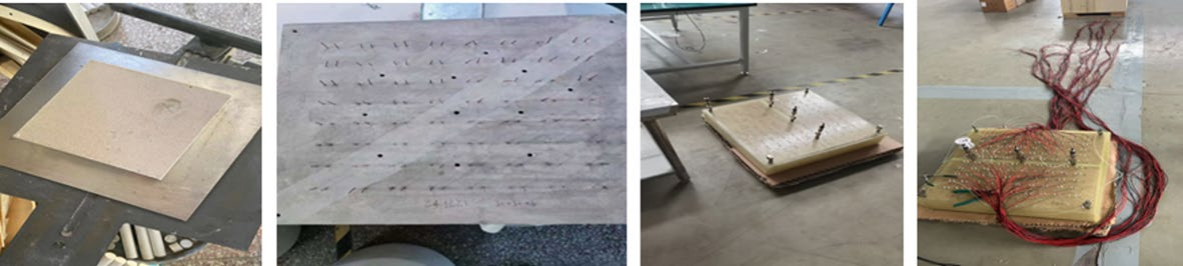
Physical model.

A three-dimensional physical simulation model composed of multiple cores was constructed based on similarity principles, simulating reservoir permeability ranges of 1000 mD(millidarcy), 3000 mD, and 5000 mD. By adopting a novel composite process of “sand-coated membrane pressurization—temperature-controlled shaping—epoxy resin encapsulation,” the heterogeneous permeability characteristics of the reservoir were effectively simulated. The model parameters were designed by referencing the dynamic and static data of the QHD32-6 block reservoir, combined with similarity principles, resulting in a three-dimensional Planar model with inter-segment interference simulation capability. In terms of well pattern configuration, the traditional pure vertical well model was overcome by introducing horizontal well pre-embedding technology, achieving efficient physical simulation of “horizontal well—vertical well” combined well patterns^13^. This provides a more realistic experimental platform for studying seepage mechanisms under complex well pattern conditions, as shown in Fig. 1^14^.

#### Geometric similarity and scaling analysis

To ensure that the physical model can equivalently represent the spatial structure and well pattern geometry of the actual reservoir, the model design in this study follows the principle of geometric similarity (All the parameters come from real data of a certain oil field in the Bohai Sea). Whereby all corresponding linear dimensions of the model and the prototype maintain a constant scaling ratio. Based on calculations of key geometric parameters, including well spacing (350 m–54 cm, 175 m–27 cm), horizontal well section length (275 m–44 cm), and formation thickness (64 m–10 cm), the representative geometric scaling factor is approximately constant, with deviations among individual parameters controlled within ± 2.4%. This indicates that the physical model exhibits good geometric similarity in terms of well pattern scale and formation thickness. On this basis, the model and the prototype satisfy the derived geometric similarity relationships, ensuring that the characteristic flow channel dimensions, interwell geometric proportions, and boundary constraints remain consistent under scaled conditions. This provides a solid geometric foundation for subsequent comparison and validation between physical model simulations and actual reservoir displacement behavior (Table 1).

**Table 1:** Key reservoir parameters of the Bohai Offshore Oilfield.

On- site parameters	Numeric value	Nnit	Experiment parameters	Numeric value	Unit
Well spacing	350	m	Well spacing	54	cm
Row spacing	175	m	Row spacing	27	cm
Horizontal well length	275	m	Horizontal well length	44	cm
Thickness	64	m	Thickness	10	cm
Porosity	0.32		Porosity	0.32	
Formation temperature	65	°C	Formation temperature	65	°C
Crude oil viscosity	200	mPa·s	Crude oil viscosity	200	mPa·s
Horizontal well depth	Upper part		Horizontal well depth	4	cm
Input rate	200	m^3^/d	Input rate	0.5	ml/min

#### Preferred experimental fluid

The experiment utilized simulated crude oil and mineralized water to investigate fluid behavior during waterflooding processes. The viscosity and density of the injected fluids were optimized to match those of the Bohai Oilfield, ensuring the representativeness and reliability of experimental results. By selecting appropriate fluids, the study could more accurately simulate real-world waterflooding operations, thereby enhancing both the rigor of the experiments and the practical value of the data obtained.

#### Experimental procedure design

The experiment steps are as follows^14^:

1. Model saturated water.

The vertically positioned sand-filled sealed model was filled with water to achieve saturation, simulating the initial water distribution in geological formations. Real-time monitoring of electrode resistance was conducted until water overflowed from the top outlet. After the resistance values stabilized at all electrodes, the bottom water injection valve was closed. Key parameters during the saturation process—including injected water volume, displacement pressure, and outlet flow rate—were recorded. These data will be utilized for subsequent experimental analysis and computational modeling.

The experimental setup measures 60 cm × 60 cm × 10 cm, with a saturation water injection rate of 10 mL/min.

2. Model saturated oil.

The model was vertically positioned with experimental oil injected at a low flow rate from the top, while water was discharged through the upper outlet. By leveraging gravity-induced oil-water-gas separation, the oil-water interface was allowed to slowly and uniformly advance downward (injecting oil at controlled rates and pressures to ensure accuracy and safety). For the 60 cm × 60 cm × 10 cm three-dimensional model, the experiment set the saturation oil injection rate at 2 ml/min until all drainage ports ceased water discharge. The injection was then stopped, the outlet valves closed, and the injected oil volume measured. To achieve full oil saturation, the model was left undisturbed for over 8 h after the initial injection halt, followed by re-saturation through top-level injection. This process was repeated multiple times until resistivity distribution indicated homogeneity.

3. Waterflooding.

The horizontal placement model conducts waterflooding experiments at a designed injection rate of 0.5 mL/min. per well. It measures and records the resistivity at electrode points (which represents the resistivity of the oil-water mixture), determines the resistivity of water and oil separately, analyzes the variation patterns of resistivity after mixing, calculates the oil-water ratio, and assesses the oil saturation level. This analysis evaluates the distribution of remaining oil across different positions in the model.

After reaching 3PV (pore volume)of water injection, close the high-permeability section outlet while maintaining production from the medium and low-permeability sections, and continue displacement until the experiment concludes at 5PV. During the experiment, data should be collected promptly at critical time points including the water injection initiation, water cut reaching 98%, and other key milestones. Based on measured oil and water production volumes, calculate parameters such as oil recovery rate, water cut, and recovery degree.

**Fig. 2:**
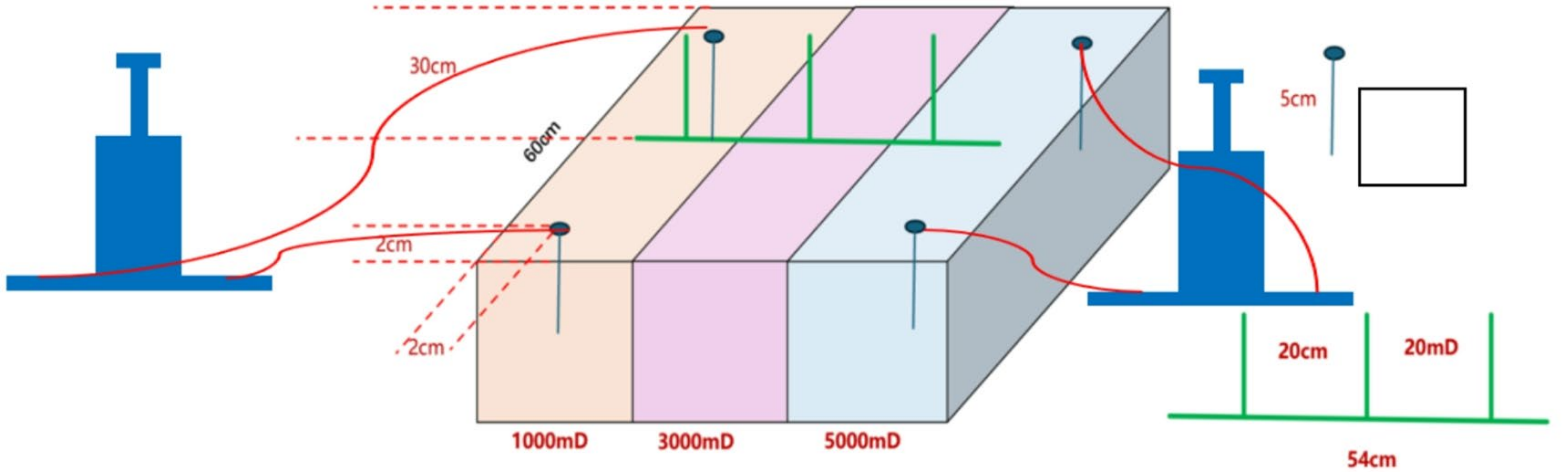
Schematic diagram of the three-dimensional physical model experiment.

Two pumps are used to inject water simultaneously. To ensure the same injection speed, each pump can only inject into the same injection well in the same seepage layer. (See Fig. 2 for the injection method).

The experimental data collection was conducted in two phases. Phase 1: After completing core oil saturation analysis, water injection experiments were performed at a constant rate of 0.5 mL/min into all injection wells. The system’s constant temperature chamber maintained a consistent temperature of 65 °C. The experiment was paused when the cumulative injection volume reached 3 PV. During this phase, data was recorded at critical time points including every 0.2 PV injection interval, water detection moments, and when the water cut reached 98% for subsequent analysis. Phase 2: After shutting down the production wells in high-permeability segments, the water drive experiment was restarted. The injection rate and temperature conditions remained unchanged, continuing until the cumulative injection volume reached 5 PV, after which the entire experiment concluded. Data were recorded at the same critical time points as Phase 1, including every 0.2 PV injection interval and when the water cut reached 98% (Table 2).

**Table 2:** Experimental step schedule.

Experimental procedure	Time
Experiment preparation	3 days
Saturation water	72 h (3 days)
Saturate with oil	120 h (5 days)
Water-driven 0-3PV	300 h (about 13 days)
Water drive 3PV~5PV (high permeability section closed)	200 h (about 9 days)

#### Experimental facility selection and experimental platform construction

**Fig. 3:**
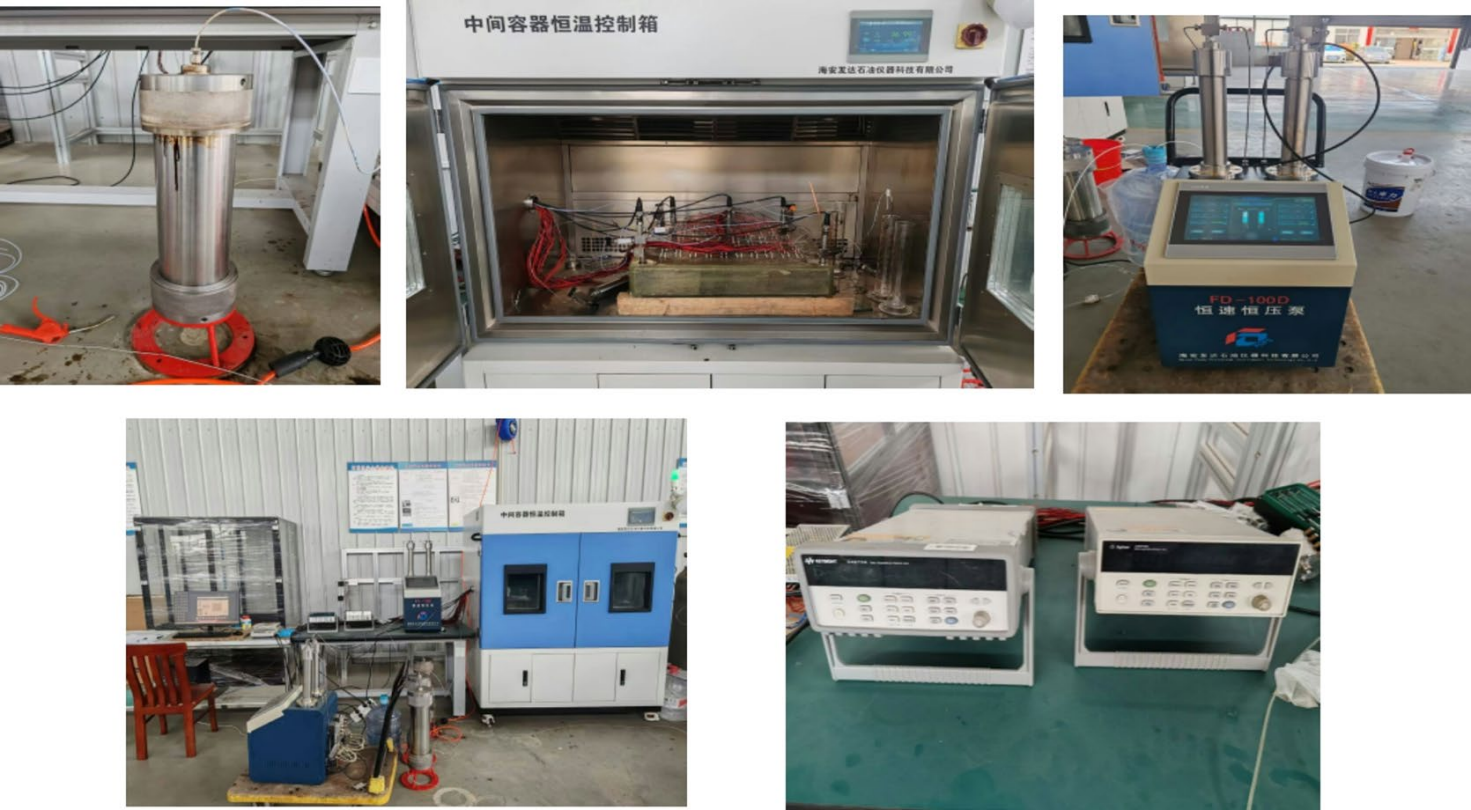
Experimental setup for the three-dimensional physical model.

The experimental system is mainly composed of 3D plate model, constant speed and constant pressure pump, saturation detection system, resistivity/pressure/oil/water acquisition system, sensor, constant temperature box, etc., as shown in Fig. 3.

#### Experimental control scheme design

**Fig. 4:**
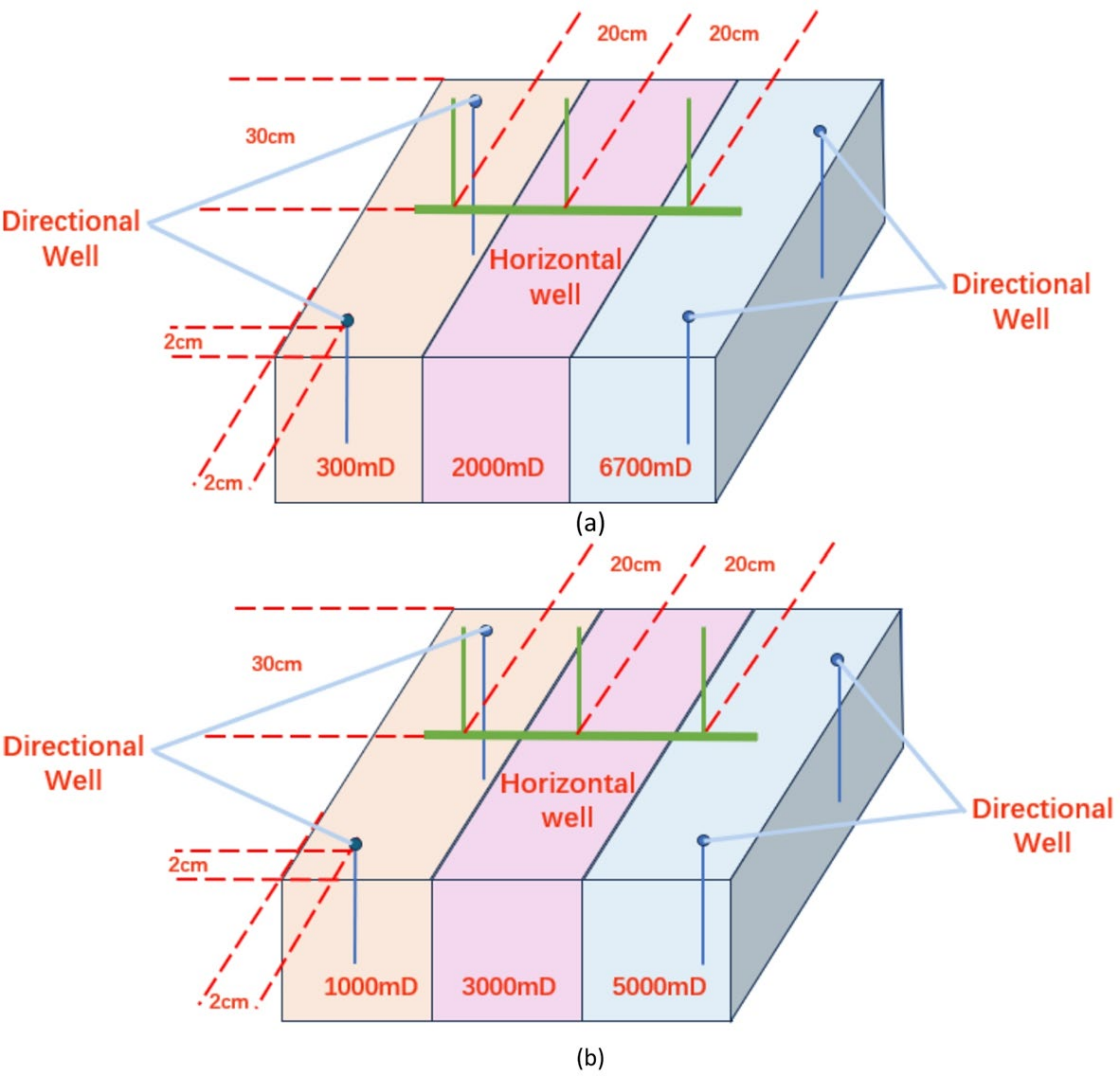
Model diagram. (a) Model diagram of scheme 1, (b) Model diagram of scheme 2.

Based on the QHD32-6 well pattern configuration, reservoir properties, and crude oil viscosity parameters, two experimental schemes were designed^10^. The crude oil used in the experiments had a viscosity of 200 mPa·s and an average permeability of 3000 mD. Scheme 1 featured a permeability gradient of 1.67/3/5 (Fig. 4a), while Scheme 2 had a gradient of 3.35/6.67/22.3 (Fig. 4b). The injection rate per well was set at 0.5 mL/min, with displacement continuing until 3 PV before stopping and shutting down the production wells in the high-permeability segments. Subsequently, displacement was maintained until 5 PV, marking the conclusion of the experiment.

#### Experimental feasibility verification

By injecting into the monitoring facility, the oil/water injection volume is recorded in real time, while the resistivity detector simultaneously records the electrical data.

The resistivity was obtained, and the variation relationship between resistivity and oil saturation was obtained.

Based on the relevant data obtained, a determination method for calculating oil saturation is established. Oil saturation is calculated by Archie formula based on the resistivity recorded in real time by the resistivity detector:

1 $${S_O}=1 - {\left( {\frac{{a{\mathrm{*}}{R_W}}}{{{\emptyset ^m}{\mathrm{*}}{R_t}}}} \right)^{\frac{1}{n}}}$$

Where: Rt: formation resistivity; Rw: formation water resistivity at 65 °C with salinity of 10,000 mg/L, 49Ω·m; ∅: porosity; a: lithology coefficient (1); m: cementation index :2; n: saturation index : 2.

The average porosity of the 3D plate model was determined to be 31.94% by the above method. After the saturation oil experiment, the internal oil saturation of the model scheme 1 was measured to be 79.05%, and the internal oil saturation of the model scheme 2 was 78.96%. The results of the two models were relatively close, which met the experimental conditions.

### Numerical simulation

To further verify the results of physical experiments and explore the influence of more complex well network configuration on interference effects, this paper adopts numerical simulation method to simulate interference effects under different reservoir layout and water drive conditions. The key steps of numerical simulation include^15^.

#### Model establishment

To achieve quantitative evaluation of horizontal wellbore interference between sections under “Horizontal - Vertical” interference patterns and precise characterization of remaining oil distribution patterns, this study utilized a flat plate model from physical experiments. By employing numerical simulation software such as CMG and adhering to geometric similarity principles, we scaled up the physical experiment model proportionally to construct a mechanistic model that aligns with the geological characteristics of the target block QHD32-6. The key parameter comparisons between the physical experiment and the mechanistic model are as follows^16^.

**Table 3:** Comparison of key reservoir parameters between three-dimensional physical model and numerical simulation.

	Physical test	Mechanism model
Well spacing	56 cm	350 m
Row spacing	28 cm	175 m
Dynamic viscosity	200mPa·s	200mPa·s
Temperature	65 °C	65 °C
Oil saturation	0.79	0.79
Injection volume	0.5 ml/min	200 m^3^/d
Reservoir thickness	10 cm	62.5 m

Table 3 details the key parameters of the model before and after scaling. Specifically, the well spacing was scaled from 56 cm in the physical experiment to 350 m in the mechanistic model, the row spacing from 28 cm to 175 m, and the reservoir thickness from 10 cm to 62.5 m, while maintaining the original scaling ratio. Other critical parameters such as fluid viscosity (200 mPa·s), temperature (65 °C), and initial oil saturation (0.79) were preserved in the model, ensuring the similarity between the physical process and the geological prototype, which lays the foundation for the reliability of subsequent numerical simulations. Based on these considerations, the numerical model was constructed.

After completing model construction and validation, this study systematically investigates the impact of reservoir properties on development efficiency. By setting three key permeability parameters (1000 mD, 3000 mD, and 5000 mD) for the mechanistic model, we simulated waterflooding processes under a five-point well pattern^4^. Through dynamic production simulation under varying permeability conditions, we aim to quantitatively reveal the intensity of inter-segment interference and its control over remaining oil accumulation segments.

#### Numerical simulation extension-analysis of main control factors

In the simulation process, CMG simulation software was employed to model the water-drive process, incorporating Darcy’s law and multiphase flow equations. Factors such as reservoir heterogeneity, permeability gradients, injection-production pressure differences, and viscosity variations were considered to simulate oil-water distribution and remaining oil enrichment during water-drive, enabling precise analysis of water-drive effects under various reservoir conditions.

Horizontal well logging operational patterns and their impact on production capacity: Permeability analysis. Using a mechanistic model to investigate dominant interference factors, we quantitatively analyzed permeability differentials and selected five levels (1.5, 2, 3, 4, 5) with average permeability of 3000mD (five data groups). The inter-segment interference numerical simulation analysis is detailed in Table 4.

**Table 4:** Different permeability contrast levels.

Permeability contrast gap	Low-permeability segment	High-permeability segment
1.5	2400 mD	3600 mD
2	2000 mD	4000 mD
3	1500 mD	4500 mD
4	1200 mD	4800 mD
5	1000 mD	5000 mD

Horizontal well section utilization patterns and their impact on production capacity: Considering water cut as a key interference factor in mechanistic model studies, we conducted quantitative analysis of water cut variations. The selected water cut range of 1.05–1.25 (with an average water cut of 85% in both low and high water cut sections across five data groups) was used for Inter-segment Interference numerical simulation analysis, as shown in Table 5.

**Table 5:** High and low water cut levels at different water cut grades.

Water cut range	Low water content section	High water content section
1.05	83%	87%
1.10	81%	89%
1.15	79%	91%
1.2	77%	93%
1.25	76%	94%

Effect of Injection-Production Pressure Difference: Using a mechanistic model, this study identifies key interference factors, with a focus on the injection-production pressure difference. A pressure differential range of 1.5 to 3.5 was selected for simulating inter-segment interference, with five data groups representing an average differential of 2 MPa across low- and high-pressure segments. The detailed setup is provided in Table 6.

**Table 6:** High and low injection-production pressure differentials at different pressure differentials.

Injection-production pressure difference	Low production pressure difference section	High injection-production pressure difference section
1.5	1.6	2.4
2	1.33	2.67
2.5	1.14	2.86
3	1	3
3.5	0.89	3.11

Horizontal well logging patterns and their impact on production capacity: Thickness analysis. Using a mechanistic model to identify key interference factors, we quantitatively analyzed thickness variations and selected the 2–6 thickness range (with five data sets covering average thicknesses of 30 m for both thin and thick sections). The inter-segment interference simulation results are presented in Table 7.

**Table 7:** Thickness of thin and thick segments at different thickness grading levels.

Thickness difference	Thin	Thick
1.5	24	36
2	20	40
2.5	17.14	42.86
3	15	45
3.5	13.33	46.67

### Interference formula and quantitative analysis

Based on physical simulation and numerical simulation, this study establishes interference formulas to quantitatively describe the interference effect in water drive process and analyze the influence of interference on remaining oil distribution and recovery rate. The following interference formulas are adopted in this study:

2 $$q=\frac{{2\pi Kh\left( {{p_e} - {p_w}f} \right)}}{{\mu \left( {ln\left( {\frac{{a+\sqrt {{a^2} - {{\left( {L/2} \right)}^2}} }}{{L/2}}} \right)+\frac{h}{L}ln\left( {\frac{h}{{2{\tau _w}}}} \right)} \right)}}$$

formula involves: q: oil well production capacity; Kh: horizontal permeability; pe, pw: formation pressure and wellbore pressure; µ: fluid viscosity; a: semi-major axis of the elliptical seepage profile; L: horizontal well length; h: reservoir thickness; rw: wellbore radius. This equation describes the coexistence of oil and water phases during water flooding processes through Darcy’s law, particularly considering factors like permeability, pressure difference, and fluid viscosity during ultra-high water cut periods. As oil fields enter the high water cut development stage, single-phase production q is replaced by oil production Qo, where Qo = total liquid volume × (1-water cut). For the proposed pressure function ψ: Since variations in oil/water viscosity and volume coefficient pressure make direct application of single-phase “pressure difference” impractical, a modified pressure function is introduced

3 $$\psi =\smallint \left( {\frac{{{K_{ro}}}}{{{\mu _o}{B_o}}}+\frac{{{K_{rw}}}}{{{\mu _w}{B_w}}}} \right)dp$$

4 $${\hat {\psi }_1}=\smallint _{0}^{{{p_1}}}\left( {\frac{{{K_{ro}}}}{{{\mu _o}{B_o}}}+\frac{{{K_{rw}}}}{{{\mu _w}{B_w}}}} \right){\mathrm{dp}}$$

5 $${\psi _w}=\smallint _{0}^{{{P_W}}}\left( {\frac{{{{\boldsymbol{K}}_{{\mathrm{ro}}}}}}{{{\mu _o}{{\boldsymbol{B}}_o}}}+\frac{{{{\boldsymbol{K}}_{{\mathrm{rw}}}}}}{{{\mu _w}{{\boldsymbol{B}}_w}}}} \right){\mathrm{dp}}$$

The parameters are defined as follows: ψ: Total function describing oil-water flow resistance, quantifying permeability and viscosity variations under different pressures; Kro: Oil phase permeability (unit: mD); Krw: Water phase permeability (unit: mD); µ: Fluid viscosity (unit: mPa s); Bo: Oil volume coefficient; Bw: Water volume coefficient; p: Pressure (unit: Pa); pf: Final maximum pressure value, indicating water pressure fluctuations in the fluid. Building on orthogonal analysis, this study derives a novel interference formula through statistical and mathematical modeling, integrating key factors and influence weights identified via orthogonal analysis. This approach enables quantitative characterization of interference effects during waterflooding processes. The new formula is presented as follows:

6 $${\mathcal{Q}_o}=\frac{{2\pi {K_n}\left[ {1 - \left( {0.312+0.102\beta {k^{\frac{3}{2}}}+0.654\beta {S_w}+0.003\beta h - 0.128\beta \Delta {P^{1.4}}} \right)} \right]h \cdot \left( {{\psi _1} - {\psi _w}} \right)}}{{\left( {{\boldsymbol{l}}{\boldsymbol{n}}\frac{{a+\sqrt {{a^2} - {{(L/2)}^2}} }}{{L/2}}+\frac{h}{L}{\boldsymbol{l}}{\boldsymbol{n}}\frac{{2h}}{{\pi {r_w}}}} \right)\frac{1}{{\left( {1 - {f_w}} \right)}}}}$$

Among them, q: oil well production capacity, representing the oil output under specific conditions. µ: fluid viscosity (unit: mPa·s), describing the internal friction force of the fluid, affecting oil-water flow. K_h_: horizontal permeability (unit: mD), describing the rock formation’s permeability in the horizontal direction. pe: formation pressure (unit: Pa), indicating the pressure within the reservoir. p w: wellbore pressure (unit: Pa), representing the pressure at the oil well. a: semi-major axis of the seepage ellipse (unit: m), indicating the maximum extent of water flow expansion. L: horizontal well length (unit: m), representing the total length of the well. h: reservoir thickness (unit: m), wellbore radius: r_w_ (unit: m), affecting fluid viscosity. fw: water cut, representing the proportion of water in the reservoir. ψ_1_: formation pseudo-pressure, describing the pressure characteristics of water phase flow. ψ_w_: water pseudo-pressure, describing the flow characteristics of water in the reservoir. α: inter-segment interference weighting degree; β: correlation coefficient; K_h_: original permeability; K((eff), h): effective permeability.

7 $$q\left( x \right)=\frac{{0.543K{K_{{\mathrm{ro}}}}\left[ {{p_e} - {p_w}\left( x \right)} \right]}}{{{\mu _0}{B_0}\left( {\ln \frac{{4h}}{{\pi {r_w}}}+lntan\frac{{\pi {z_w}}}{{2h}}} \right)}}$$

Where: q(x): production rate per unit length (oil phase production); Kr: oil phase permeability, indicating the oil’s ability to flow through rock; µ: fluid viscosity, affecting flow resistance; Bo: oil’s volume coefficient; h: reservoir thickness; r_w_: wellbore radius (m); x: horizontal distance between wells; π: pi.

According to the production formula of microsegment, the oil production index of a single well can be expressed by the following formula:

8 $${J_{doi}}=\frac{{0.543 \cdot Ki \cdot Kroi}}{{\mu oBo\left( {ln\frac{{4h}}{{\pi rw}}+lntan\frac{{\pi zw}}{{2h}}} \right)}}$$

Where: q(x): production rate per unit length (oil phase production); Kr: oil phase permeability, indicating the oil’s ability to flow through rock; µ: fluid viscosity, affecting flow resistance; Bo: oil’s volume coefficient; h: reservoir thickness; r_w_: wellbore radius (m); x: horizontal distance between wells; π: pi.

The mathematical expression of the interference system is:

9 $${\eta _ \circ }=\frac{{\mathop \sum \limits^{{\mathop {i=1}\limits_{n} }} {J_{{\mathrm{doi}}}} - {J_{{\mathrm{ho}}}}}}{{\mathop \sum \limits^{{\mathop {i=1}\limits_{n} }} {J_{{\mathrm{doi}}}}}}$$

Where: η_o_: The interference coefficient between horizontal well sections, quantifying the interference level between different sections during water flooding; J_doi_: The oil production index of each micro-element section; Jho: The oil production index of the original well section.

Finally, this study establishes a comprehensive interference formula for analyzing and predicting interference effects in water drive process by combining all derivation processes and experimental results. The formula is as follows:

10 $${\eta _o}=1 - \frac{{\left( {1 - {f_w}} \right) \cdot 2\pi {K_h}(0.688+0.102,\beta {k^{3/2}}+0.654\beta {S_w}+0.003,\beta h - 0.128\beta \left( {\Delta F{)^{14}}} \right)h\left( {{\psi _1} - {\psi _w}} \right)}}{{\mathop \sum \limits^{{\mathop {i=1}\limits_{n} }} \frac{{0.543{K_i}{K_{wi}}}}{{{\mu _o}{B_o}\left( {\ln \frac{{4h}}{{\pi {r_w}}}+lntan\frac{{\pi {z_{wi}}}}{{2h}}} \right)}}\left( {\ln \frac{{\alpha +\sqrt {{\alpha ^2} - {{(L/2)}^2}} }}{{L/2}}+\frac{{{k_1}}}{L}\ln \frac{{2h}}{{\pi {r_w}}}} \right) \cdot \left( {{p_e} - {p_w}\left( x \right)} \right)}}$$

Where: Q_o_: Revised well production capacity; ψ_1_, ψ_w_: Designated pressures, describing how pressure differentials affect water flow during water flooding; a, L: Semi-major axis of the elliptical seepage profile and horizontal well length.

### Analysis of main control factors

In this study, to thoroughly investigate the key factors influencing waterflooding processes under planar straight interference patterns, this section systematically analyzes the mechanisms of permeability, viscosity, injection-production pressure difference, and formation thickness as primary control factors through experimental and simulation setups. The specific research methodology is outlined below:

Analysis of Permeability Variation Effects: To investigate how permeability differences influence interference during waterflooding, we established distinct permeability zones in both physical and numerical simulations.In the physical models, core layers with contrasting permeability values were constructed to simulate interference between high- and low-permeability regions. In the numerical simulations, permeability gradients were incorporated into flow models to analyze their impact on sweep efficiency. This systematic approach allowed us to evaluate how variations in permeability govern interference between injection and production segments.

Viscosity Variation: To investigate viscosity’s influence on waterflooding efficiency, this study adopted fluids with varying viscosities under different simulation conditions. Through physical experiments using crude oil and mineralized water similar to those in actual reservoirs, we simulated fluid flow behavior in different reservoir segments. The analysis focused on how fluid mobility with different viscosities affects water displacement efficiency in various permeability segments. In numerical simulations, we further examined the impact of viscosity variations and fluid flow characteristics on interference effects and uneven waterflood sweep.

Injection-production pressure difference: Variations in injection-production pressure difference are a critical factor influencing interference effects during waterflooding processes. This study analyzes the impact of inter-segment interference through controlled variations in pressure differentials. Through physical simulation experiments, precise regulation of injection-production flow rates and pressure differentials was adopted to investigate how high-pressure differentials affect water flow paths and water drive coverage. Numerical simulations further validated the effects of pressure differentials on water drive efficiency and interference intensity, particularly examining breakthrough phenomena under high-pressure differential conditions and their impacts on different permeability segments.

Layer thickness variation: The thickness of oil reservoir layers significantly affects waterflooding efficiency, particularly in formations with substantial thickness differences. This study adopted physical modeling to simulate waterflooding processes under varying thickness conditions, with a focus on analyzing the sensitivity of thin-layer sections. Through numerical simulations with different thickness configurations, we investigated water distribution patterns, interference intensity, and their impacts on remaining oil distribution.

Through parameter configuration and analysis, this study not only reveals the mechanisms of various factors affecting interference effects, but also provides a basis for optimizing well pattern layout and adjusting injection-production flowlines in oilfield development. These methods can effectively enhance waterflooding efficiency, optimize development strategies for ultra-high water-cut oilfields, and particularly in complex heterogeneous reservoirs, help improve the recovery of remaining oil.

## Results and discussion

This study systematically investigates the impact of the “Horizontal - Vertical” interference pattern on remaining oil enrichment patterns in Bohai Oilfield during the ultra-high water cut period by combining physical simulation and numerical simulation. Through physical simulation experiments and numerical simulation analysis, the following main results are obtained:

### Analysis of physical experiment results

The physical simulation experiment investigated intra-layer inter-segment interference effects during waterflooding by configuring parameters like permeability and injection-production pressure differentials in a 3D physical model. The methodology involved injecting simulated crude oil and mineralized water, while analyzing water displacement efficiency across different volume ranges (0PV-3PV, 3PV-5PV) by precisely controlling injection flow rate, pressure, and temperature.

#### Result analysis of scheme 1

**Fig. 5:**
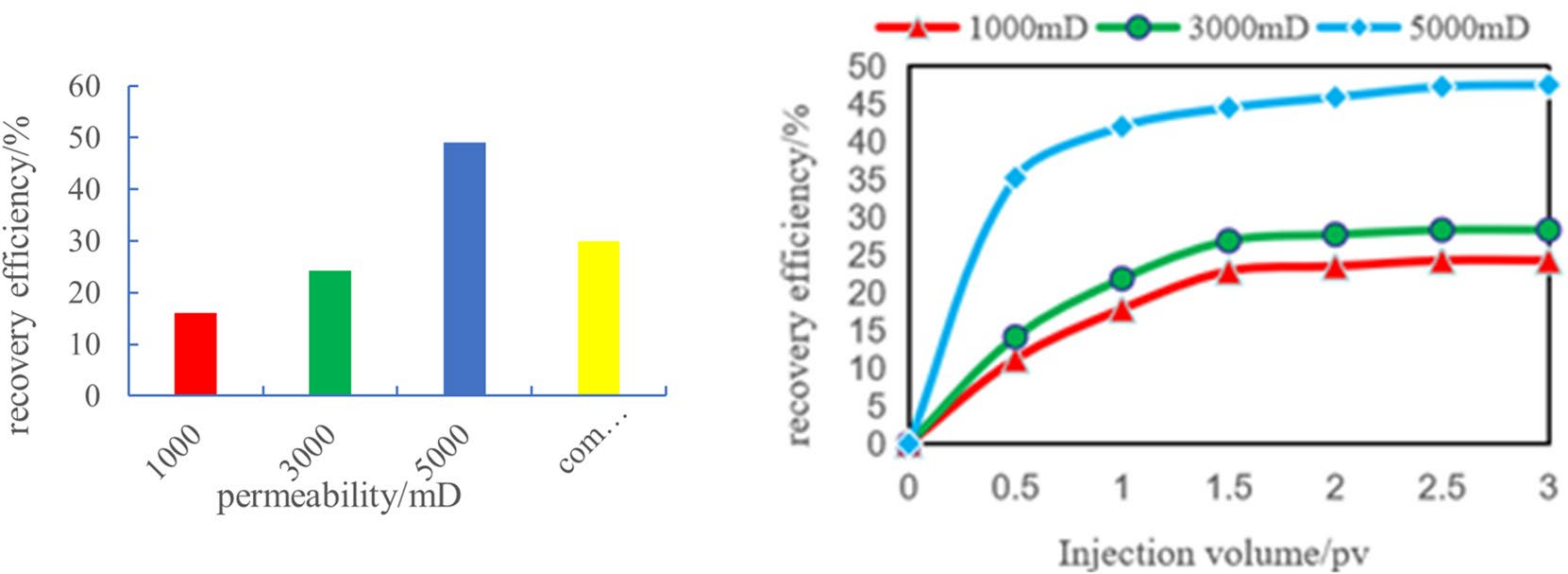
Reservoir recovery rate curve of scheme 1.

In the 0-3PV experimental scheme, significant differences in recovery factor were observed across permeability segments. The overall recovery rate reached 33.37%, with specific values of 47.53% in high-permeability segments, 28.31% in medium-permeability segments, and 24.28% in low-permeability segments. The interference coefficients were 0.48 for low-permeability segments, 0.43 for medium-permeability segments, and 0.4 for high-permeability segments. In high-permeability segments, rapid fluid penetration and expansion resulted in minimal inter-segment interference, leading to higher recovery factor. Conversely, severe inter-segment interference in low-permeability segments caused uneven fluid distribution across different segments, resulting in relatively lower recovery factor (Fig. 5).

These differences are not only caused by the characteristics of rock layers with different permeability, but more importantly, they are affected by the inter-segment interference within the formation. The difference in fluid transport path and permeability between different regions leads to the interaction between fluids in different sections, which affects the recovery rate.

**Fig. 6:**
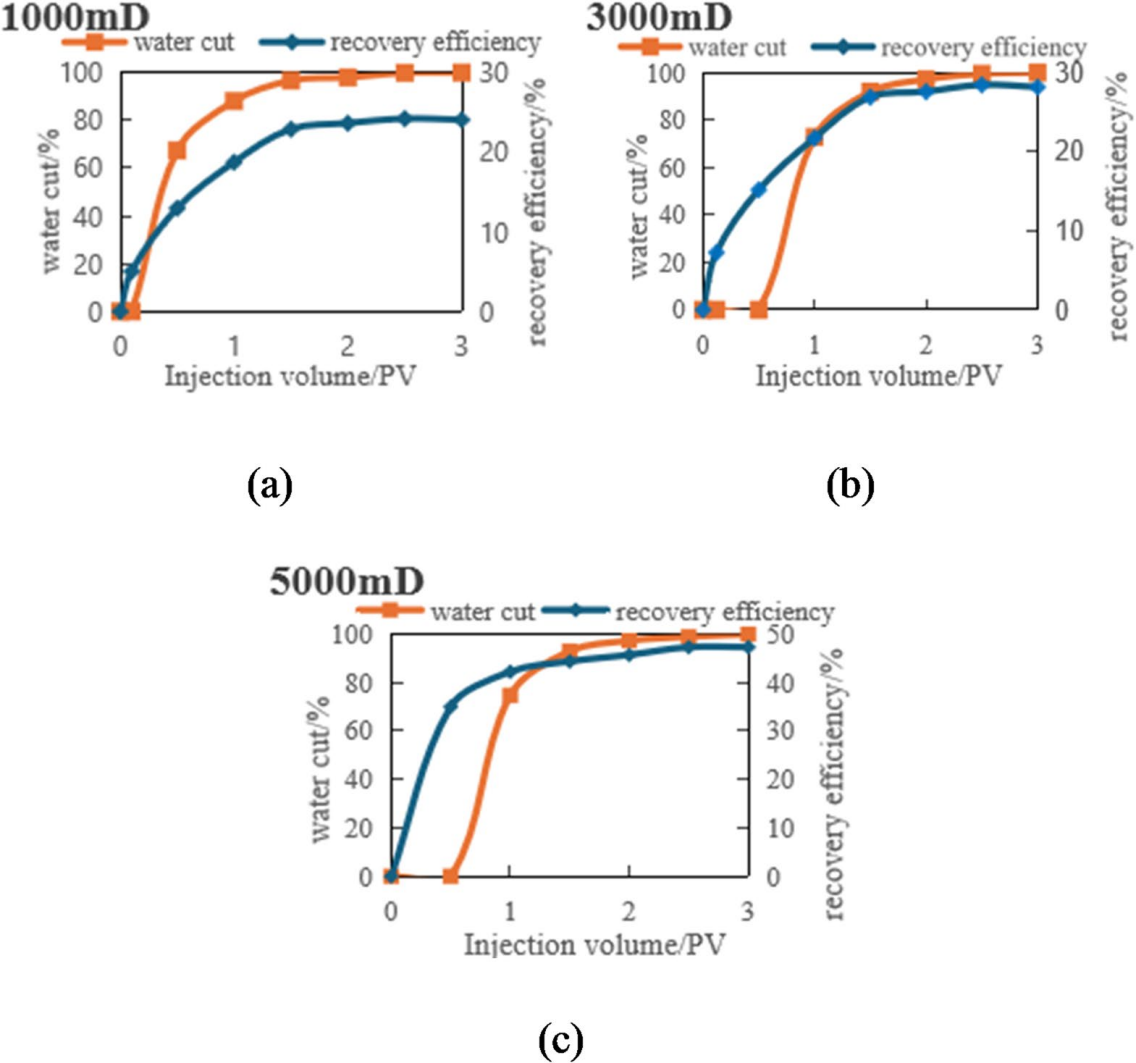
Variation of oil recovery and water cut with injection volume (0–3 PV) in different permeability segments. (a) Low-permeability segments, (b) Medium-permeability segments, (c) High-permeability segments.

In low-permeability segments (1000 mD), the rate of oil recovery increases significantly with injection volume. During the initial phase, injected fluids struggle to penetrate and expand rapidly, leading to complex fluid-rock interactions that form multiple flow channels. As injection continues, fluids gradually penetrate deeper into rock layers, intensifying inter-segment interference and slowing their propagation. Restricted by pore structure limitations, fluid pathways become obstructed, making heterogeneity’s impact on fluid expansion particularly pronounced. Consequently, oil recovery in these segments grows slowly, rarely exceeding 25%, demonstrating how inter-segment interference constrains production efficiency (Fig. 6a).

In the medium-permeability zone (3000 mD), fluid mobility improved compared to the low-permeability zone, while inter-segment interference effects were somewhat reduced. As injection rates increased, recovery factor showed a significant upward trend, but growth rates began to slow when reaching a certain threshold. Although medium-permeability segments demonstrated notable improvements over low-permeability segments, fluid propagation speeds still lagged behind high-permeability segments. With further injection increases, inter-segment interference effects became increasingly apparent—particularly at higher injection levels where fluid expansion was progressively constrained. The heterogeneity of rock pores exerted more pronounced influence on fluid flow, resulting in slower recovery rate growth. Therefore, despite medium-permeability segments achieving higher recovery factor than low-permeability segments, inter-segment interference prevented them from matching high-permeability zone performance (Fig. 6b).

In high-permeability segments (5000 mD), fluid can rapidly penetrate and expand into adjacent areas due to enhanced permeability, resulting in minimal inter-segment interference. When injection rates are low, recovery factor show a significant increase. As injection continues, recovery factor keep rising until stabilizing near 3PV, ultimately reaching 47.53% (Fig. 6c). This trend reflects the enhanced fluid mobility in high-permeability segments, which facilitates rapid fluid propagation through rock layers and reduces inter-segment interference. However, even with this advantage, increased fluid injection gradually fills pore spaces, and the persistent heterogeneity effect causes recovery rate growth to slow down. Despite low inter-segment interference, the inherent rock heterogeneity prevents high-permeability segments from achieving 100% recovery factor.

Scheme 1 (3PV-5PV): To enhance permeation efficiency, the high-permeability outlet well was shut down when the injection volume reached 3 PV.

**Fig. 7:**
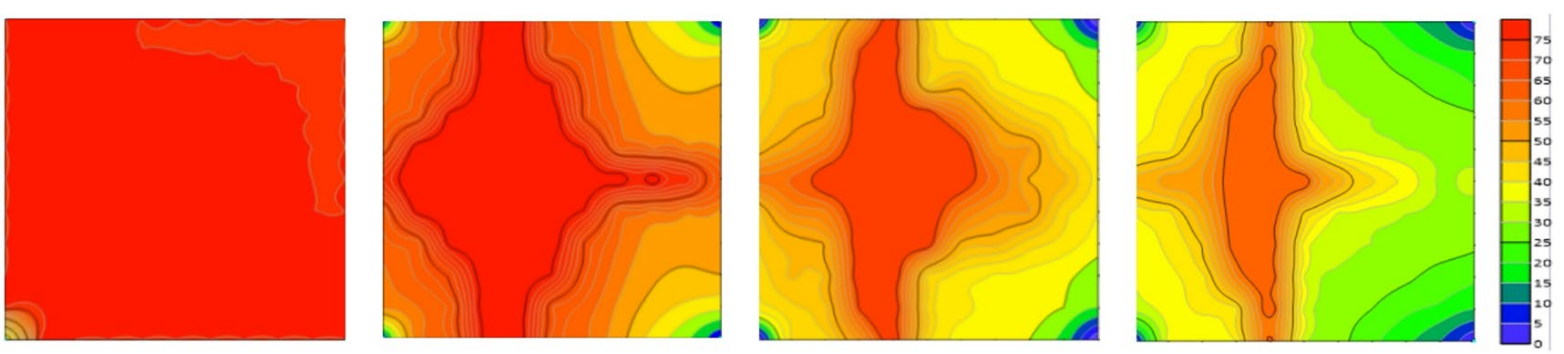
Evolution of remaining oil distribution in the model under different injection volumes (0, 0.5, 1, and 3 PV).

As shown in Fig. 7, At 0.5 PV injected, the low-permeability zone showed almost no significant oil recovery response. Remaining oil remained concentrated, forming distinct bypassed oil zones (or dead oil zones). Due to the poor permeability, fluids struggled to penetrate deeper into this zone, leaving a substantial volume of crude oil effectively unrecovered. In contrast, the high-permeability zone exhibited a favorable recovery response, where fluids rapidly swept through and displaced a significant amount of oil, markedly reducing the residual oil saturation.

As the injection volume increased to 1.0 PV, oil recovery in the high-permeability zone began to stabilize, with a substantial reduction in remaining oil. However, recovery in the low-permeability zone remained limited, with remaining oil still significant, demonstrating its inherent recovery constraints.

When the injection volume reached 3.0 PV, oil recovery in the high-permeability zone had largely stabilized, with remaining oil approaching residual saturation levels. In contrast, the low-permeability zone still showed no significant improvement in recovery. The persistent, large bypassed oil regions underscored that the recovery bottleneck in the low-permeability zone had not been overcome.

**Fig. 8:**
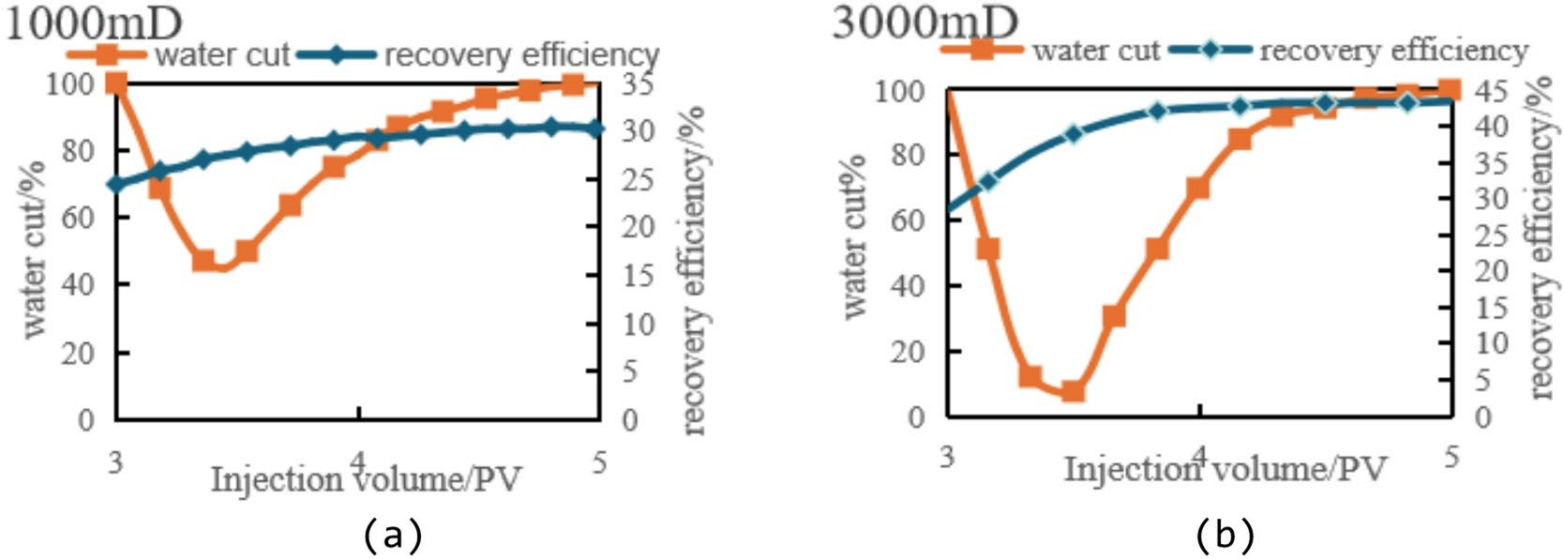
Variation of oil recovery and water cut with injection volume (3–5 PV) in different permeability segments. (a) Low-permeability segments, (b) Medium-permeability segments.

As shown in Fig. 8a the recovery rate in low-permeability segments grows slowly after reaching 3PV with increasing injection volume, approaching a plateau. The improvement in recovery rate between the 0–3PV and 3–5PV stages is minimal, with an increase of less than 6% points (from approximately 24.28% in the 0–3PV stage to 30.18% in the 3–5PV stage). This indicates poor fluid mobility in low-permeability segments, where fluids struggle to penetrate deeper rock layers effectively, resulting in limited recovery of remaining oil. Consequently, the recovery rate in these segments is constrained by inter-segment interference effects and poor fluid expansibility. Although streamline adjustments and shutting down high-permeability wells have shown some improvement, the fluid’s expansion into low-permeability segments remains insufficient.

Compared to low-permeability segments, the medium-permeability zone (Fig. 8b) demonstrates significantly higher recovery factor. During the 3–5PV phase, the rate of increase becomes particularly pronounced, with a substantial 15% point rise from 28.31% to 43.31%. Imaging analysis reveals that streamline adjustment and the closure of high-permeability production wells proved especially effective in this zone. The recovery rate in the medium-permeability zone improved by 15% points between the 0–3PV and 3–5PV phases, substantially enhancing extraction efficiency. The fluid’s excellent expansibility in this zone enables more injected fluid to drive crude oil recovery, thereby optimizing the overall production performance.

**Fig. 9:**
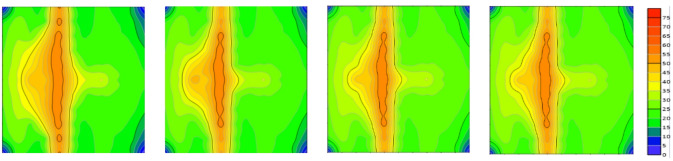
Evolution of remaining oil distribution under different injection volumes (3.5, 4.0, 4.5, and 5.0 PV).

Figure 9’s remaining oil distribution evolution sequence (3.5 PV, 4 PV, 4.5 PV, 5 PV) physically illustrates these differences. The figure clearly shows that after shutting off high-permeability channels, the displacement fluid preferentially penetrated and effectively displaced large volumes of remaining oil in the original medium-permeability segments, significantly expanding their sweep volume. In low-permeability segments, although the displacement front advanced, the internal remaining oil accumulation segments remained underutilized, with minimal color changes. This directly explains the fundamental reason for the limited increase in recovery factor.

**Fig. 10:**
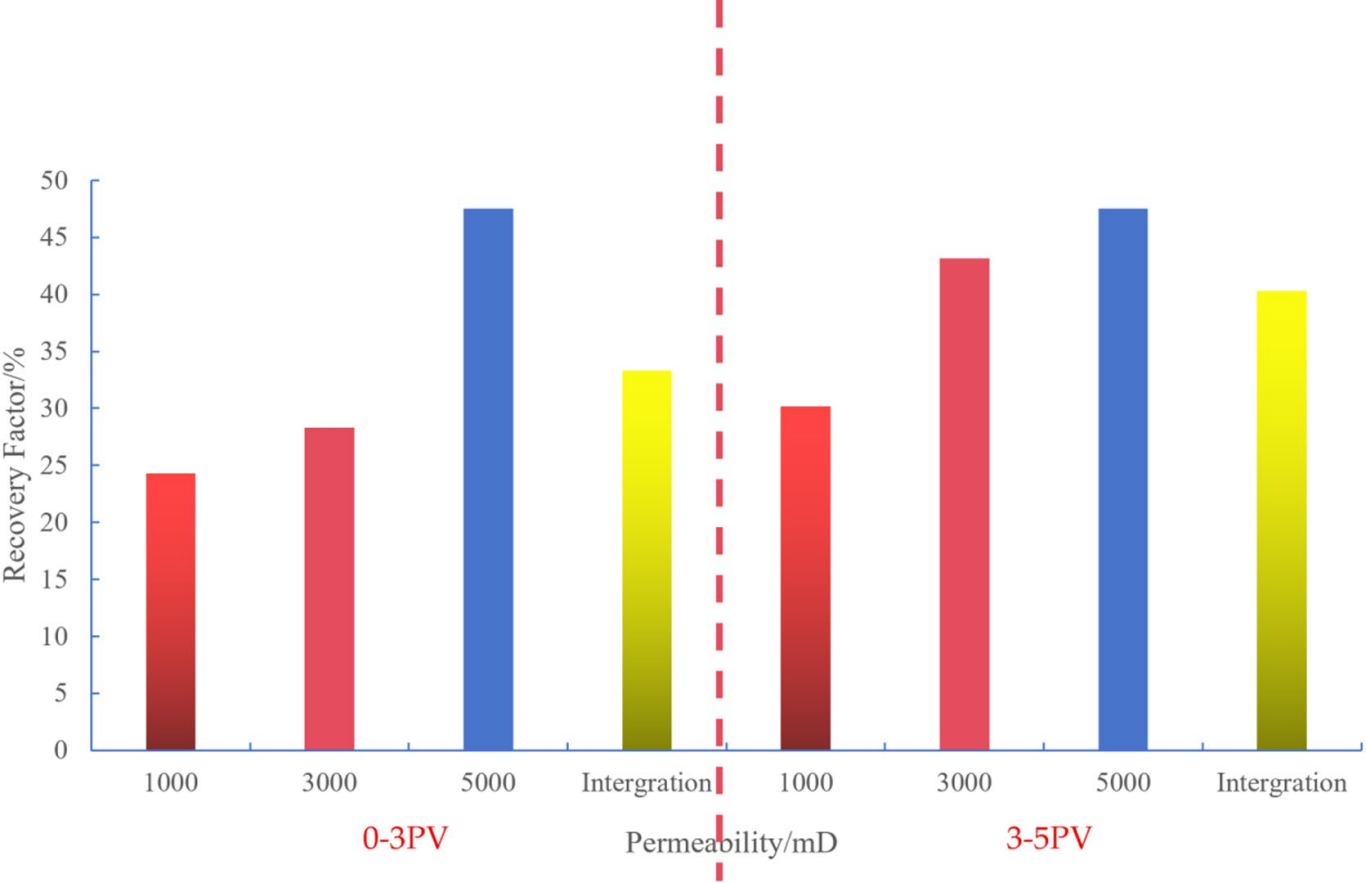
Comparison of recovery factor before and after adjustment of development scheme 1.

Figure 10’s comparative images clearly demonstrate the significant differences before and after implementing the measures. After three PV cycles, the overall recovery rate resumed a clear upward trend. Meanwhile, the recovery rate in low-permeability segments showed slower growth, while that in medium-permeability segments exhibited more pronounced increases. This proves that the adjustment measures successfully redirected subsequent injected water to underutilized medium and low-permeability segments. Closing high-permeability production wells effectively reduced the “leakage” phenomenon in high-permeability areas, redistributing fluid pressure and displacement capacity to medium-permeability segments. This measure significantly boosted the recovery rate in medium-permeability segments, achieving a 15% increase. Simultaneously, reducing preferential flow channels in high-permeability segments allowed more fluid to penetrate medium-permeability layers, thereby enhancing their recovery efficiency. Although streamline adjustment improved fluid utilization in low-permeability segments, the limited mobility and strong inter-segment interference effects in these segments resulted in relatively modest recovery rate improvements.

#### Result analysis of scheme 2

In the displacement experiment of scheme 2, the injection amount was increased from 0 PV to 3PV, and the influence of planar heterogeneity on oil displacement was evaluated by monitoring the changes of recovery rate and water content in different permeability regions.

**Fig. 11:**
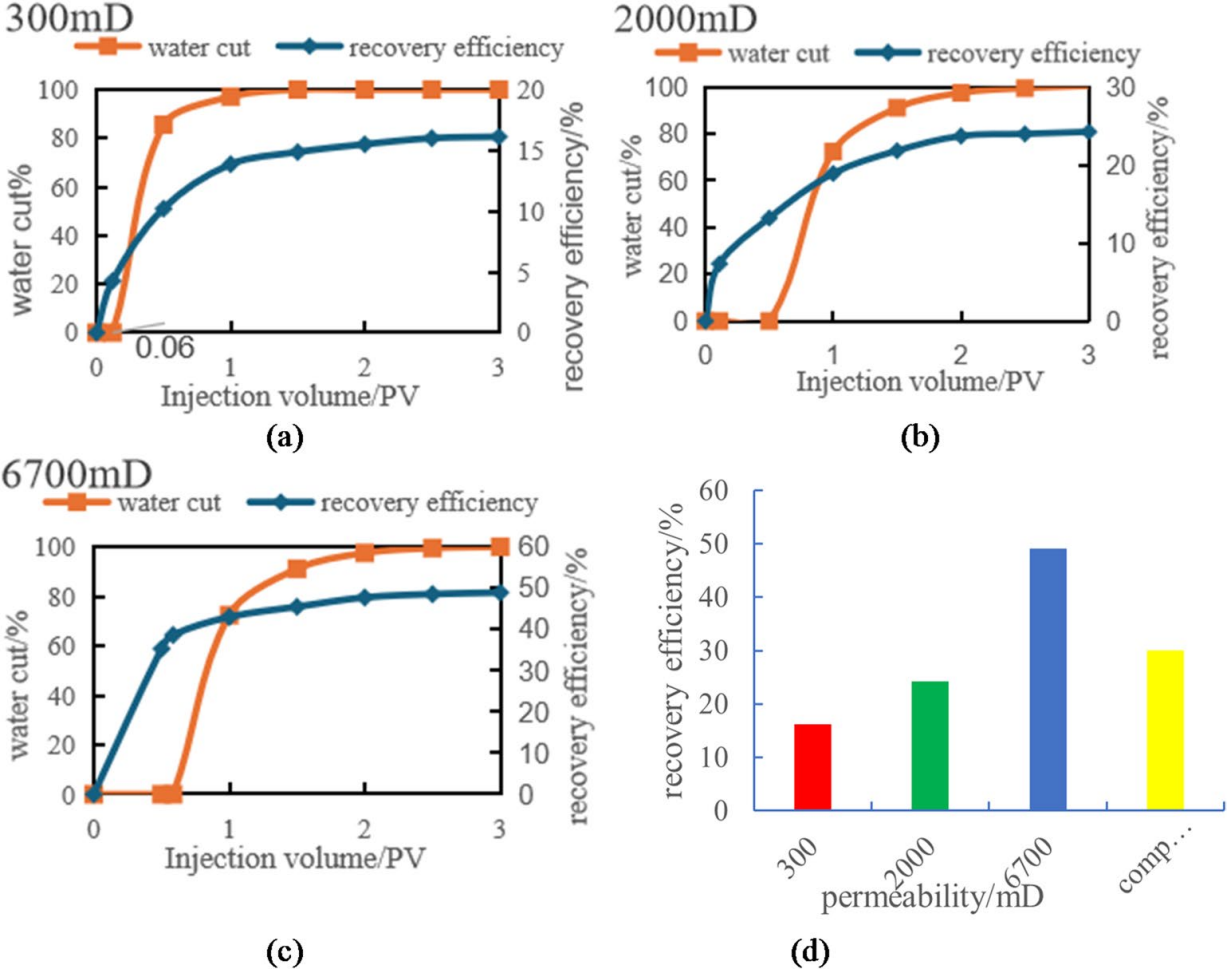
Variation of oil recovery and water cut with injection volume (0–3 PV) under different permeability segments in development scheme 2. (a) Low-permeability segments, (b) Medium-permeability segments, (c) High-permeability segments, (d) Reservoir recovery under different permeability levels.

As shown in Fig. 11a, the graph illustrating the relationship between water cut and waterflooding efficiency in low-permeability segments reveals that poor fluid permeability and limited expansibility in these areas result in slow yield enhancement. Although water cut increases slightly with injection volume, it remains at only 16.12% near 3PV, indicating poor fluid mobility and significant inter-segment interference effects. Meanwhile, water cut rises progressively as injected fluid primarily flows through high-permeability channels, leading to limited fluid expansion in low-permeability layers and restricted water displacement effectiveness, which explains the rapid water cut increase. However, the low displacement efficiency in these segments means that water cut growth fails to effectively boost recovery factor, further demonstrating the constrained recovery potential of this reservoir type.

Compared to low-permeability segments, medium-permeability segments demonstrate significantly higher recovery factor, with a marked increase starting near 1PV. Although the rate of recovery improvement gradually slows with increasing injection volume, these segments still achieve optimal recovery efficiency, reaching 24.19% (Fig. 11b). This indicates minimal inter-segment interference effects in medium-permeability segments. The water cut remains relatively stable, showing gradual increases with injection volume, demonstrating effective water displacement. The fluid’s excellent expansibility allows injected water to displace substantial crude oil, resulting in progressive water cut elevation.

Figure 11c illustrates the water cut variation in high-permeability segments. These segments exhibit superior fluid permeability, allowing rapid fluid penetration and expansion, which drives faster recovery rate growth. When injected fluid volume reaches 0.5PV, recovery factor show significant improvement. As injection increases, recovery factor continue to rise until stabilizing near 3PV, achieving 49.11% – demonstrating the most effective water displacement in high-permeability areas. Water cut initially rises sharply but stabilizes with increased injection. The exceptional fluid mobility in these segments enables rapid fluid entry into rock layers, effectively transporting large volumes of crude oil, thus accelerating water cut growth. As recovery factor stabilize, water cut levels also plateau, highlighting the high water drive efficiency characteristic of high-permeability segments.

Figure 11d quantifies the aforementioned trends and provides a comparative analysis of regional recovery factor. The diagram summarizes Scheme II’s performance across 0-3PV injection ranges, clearly demonstrating how recovery factor in high-permeability, medium-permeability, and low-permeability segments vary with injection volume. This further validates the controlling effect of Planar heterogeneity on displacement processes recovery factor were 49.11% in high-permeability segments, 24.19% in medium-permeability segments, and 16.12% in low-permeability segments, with an overall recovery rate of 30%. The high-permeability zone achieved 3.05 times the recovery rate of the low-permeability zone, highlighting significant inter-segment differences caused by Planar heterogeneity. Interference coefficient analysis revealed 0.38 for high-permeability segments, 0.47 for medium-permeability segments, and 0.52 for low-permeability segments, indicating the highest inter-segment interference level in low-permeability segments where production capacity is significantly suppressed. These findings demonstrate that Planar heterogeneity critically influences fluid displacement behavior, leading to substantial recovery rate variations across different segments.

**Fig. 12:**
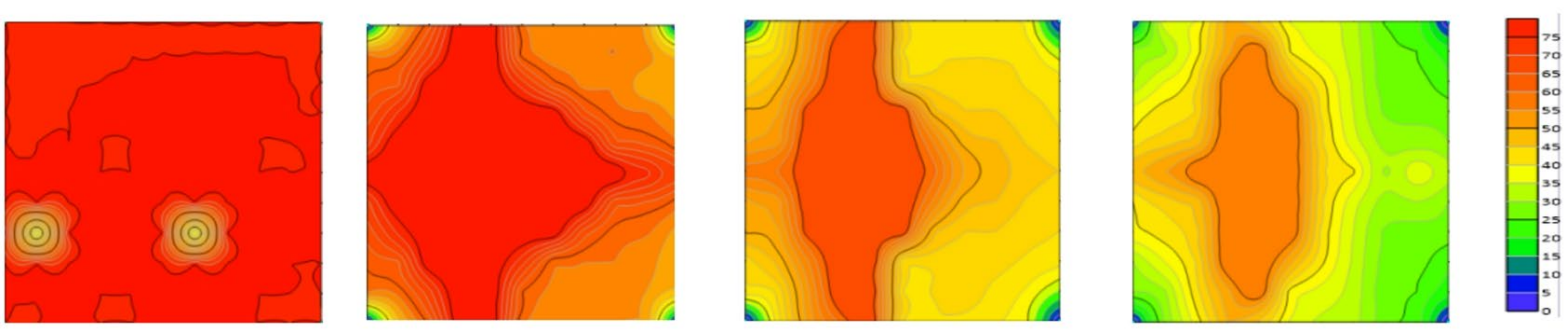
Evolution of remaining oil distribution under injection volumes of 0, 0.5, 1.0, and 3.0 PV.

The remaining oil distribution map from the fluid injection process (Fig. 12) more intuitively illustrates this trend. At 0PV: Initially, remaining oil is uniformly distributed across all segments with significant amounts remaining in each area.0.5PV: remaining oil in high-permeability segments begins to decrease markedly as fluid rapidly expands and carries away substantial crude oil. Low-permeability segments maintain relatively uniform remaining oil distribution, indicating poor water displacement efficiency. 1PV: remaining oil in high-permeability segments further diminishes while low-permeability segments retain remaining oil, demonstrating weaker displacement effectiveness. Medium-permeability segments show reduced remaining oil but fail to achieve rapid displacement like high-permeability segments. 3PV: High-permeability segments exhibit almost no remaining oil, while low-permeability segments retain significant undisplaced oil, forming distinct Unswept segments. remaining oil distribution is closely related to inter-segment interference effects. High-permeability segments demonstrate minimal inter-segment interference due to strong fluid expansibility, enabling effective remaining oil displacement. Conversely, low-permeability segments exhibit significant inter-segment interference and poor fluid expansibility, resulting in substantial undisplaced remaining oil.

During the phase when the injection rate increased from 3 PV to 5 PV, the injection-production flowline was adjusted by shutting down the outlet well in the high-permeability area to investigate the impact of this on the displacement effect in the medium and Low-permeability segments.

**Fig. 13:**
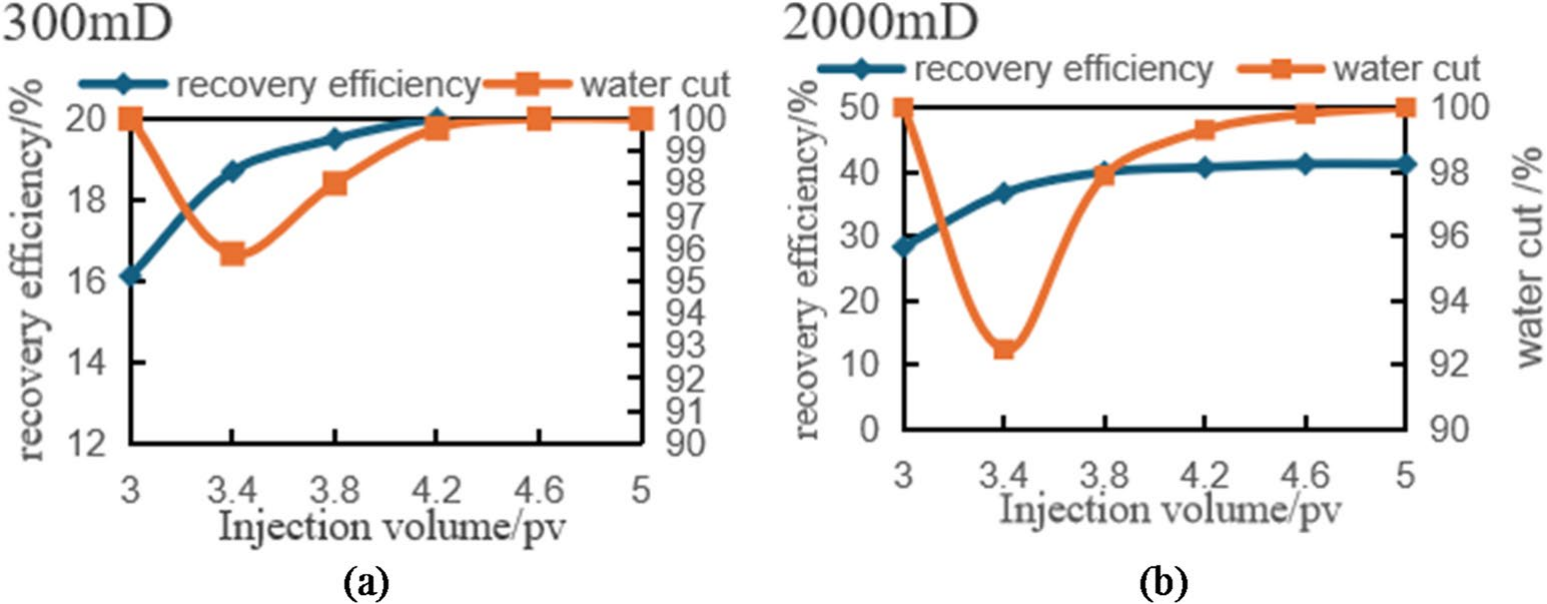
Variation of oil recovery and water cut with injection volume (3–5 PV) in low- and medium-permeability segments. (a) Low-permeability segments, (b) Medium-permeability segments.

As shown in Fig. 13a, during the 3-5PV injection period, the low-permeability zone maintained a sluggish recovery rate increase of merely 4% points, while the water cut remained persistently high. The inter-segment interference effect in permeable segments proved most pronounced, with poor fluid mobility severely limiting recovery rate enhancement. Even with increased injection volumes, the low-permeability zone showed minimal recovery rate growth, indicating suboptimal water displacement efficiency. The inter-segment interference effect prevented effective fluid penetration into deeper reservoir layers, resulting in ineffective displacement of remaining oil.

Figure 13b illustrates the dynamic changes in the medium-permeability segments during the same period. The data reveals a notable 12.9% increase in oil recovery rate, with water cut showing a more moderate trend, indicating effective enhancement of reservoir development. Although inter-segment interference remains significant in permeable segments, it is less pronounced compared to low-permeability areas. Following streamline optimization, the medium-permeability zone achieved a 12.9% recovery rate improvement, demonstrating enhanced fluid penetration into deeper layers. This optimization alleviated inter-segment interference effects and significantly boosted recovery efficiency.

**Fig. 14:**
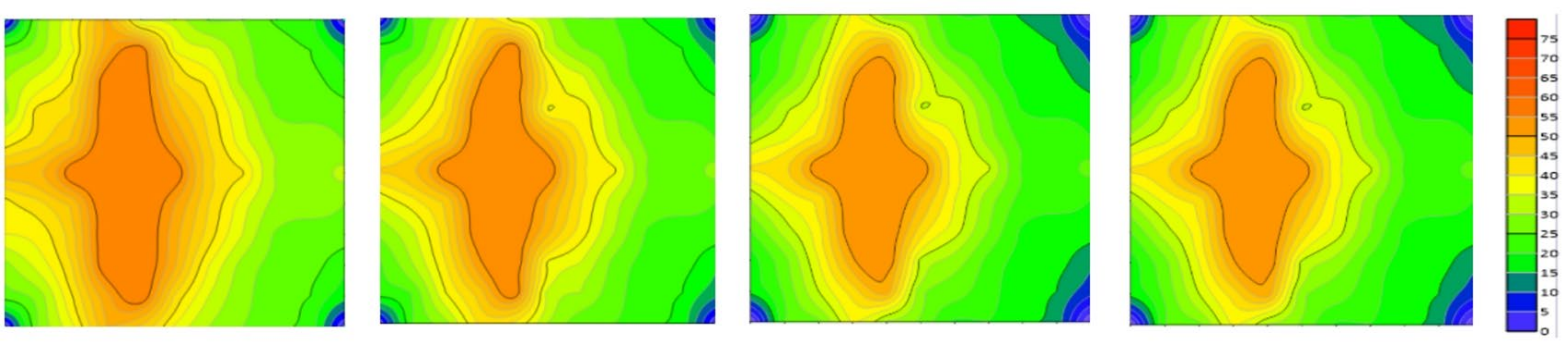
Evolution of remaining oil distribution in the three-dimensional physical model under injection volumes of 3.5, 4.0, 4.5, and 5.0 PV.

This phenomenon is visually demonstrated in the remaining oil distribution map (Fig. 14) for the 3PV-5PV reservoir. When the high-permeability outlet well is shut down, the fluid flow path is forced to redistribute, redirecting the displacement front from the original high-permeability channel to the medium and low-permeability segments. This adjustment effectively mitigates the “short-circuit” effect of high-permeability layers on fluid flow, thereby enhancing the utilization of medium-permeability segments. However, due to significant inter-layer permeability variations, inter-segment interference persists, particularly evident in low-permeability areas.

Significant improvements were observed in the medium-permeability zone: After shutting down the high-permeability well, the restricted injection water flow into the high-permeability layer redirected more displacement energy to the medium-permeability zone, resulting in a more uniform sweep volume. As shown in the figure, the remaining oil in the medium-permeability zone during the 3.5–5PV stage decreased markedly, with enhanced reservoir utilization rates, validating the positive effects of streamline adjustment. The low-permeability zone remained constrained: Although optimized injection-production configurations improved displacement pressure gradients, the complex pore structure and limited fluid flow channels in low-permeability segments, coupled with persistent inter-segment interference effects, hindered fluid movement. The displacement front advanced slowly, with remaining oil remaining concentrated on both sides of the low-permeability zone, exhibiting the characteristic “un-swept dead oil zone” pattern.

**Fig. 15:**
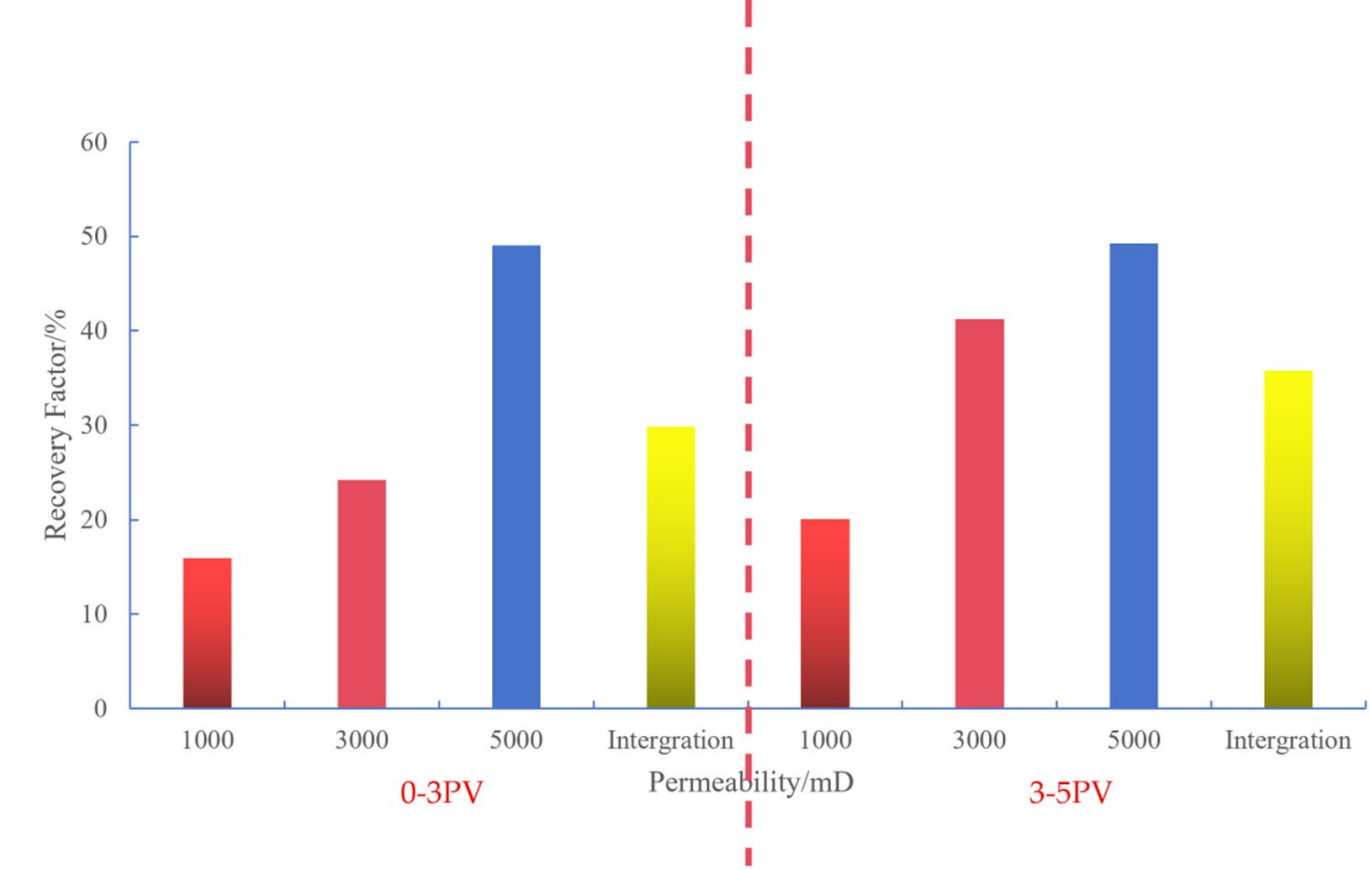
Comparison of reservoir recovery factor before and after injection-production flowline adjustment in scheme 2 (0–3 PV vs. 3–5 PV).

To comprehensively evaluate the effectiveness of streamline adjustment, Fig. 15 compares the changes in recovery factor between two phases (0-3PV and 3-5PV) of Scheme II before and after the adjustment. The figure clearly demonstrates that streamline adjustment achieves the most significant increase in recovery factor in medium-permeability segments, while the contribution rate in low-permeability segments remains the lowest. This further confirms the controlling effect of Planar heterogeneity on displacement processes and highlights the limitations of streamline adjustment measures.

#### Comprehensive analysis results

**Fig. 16:**
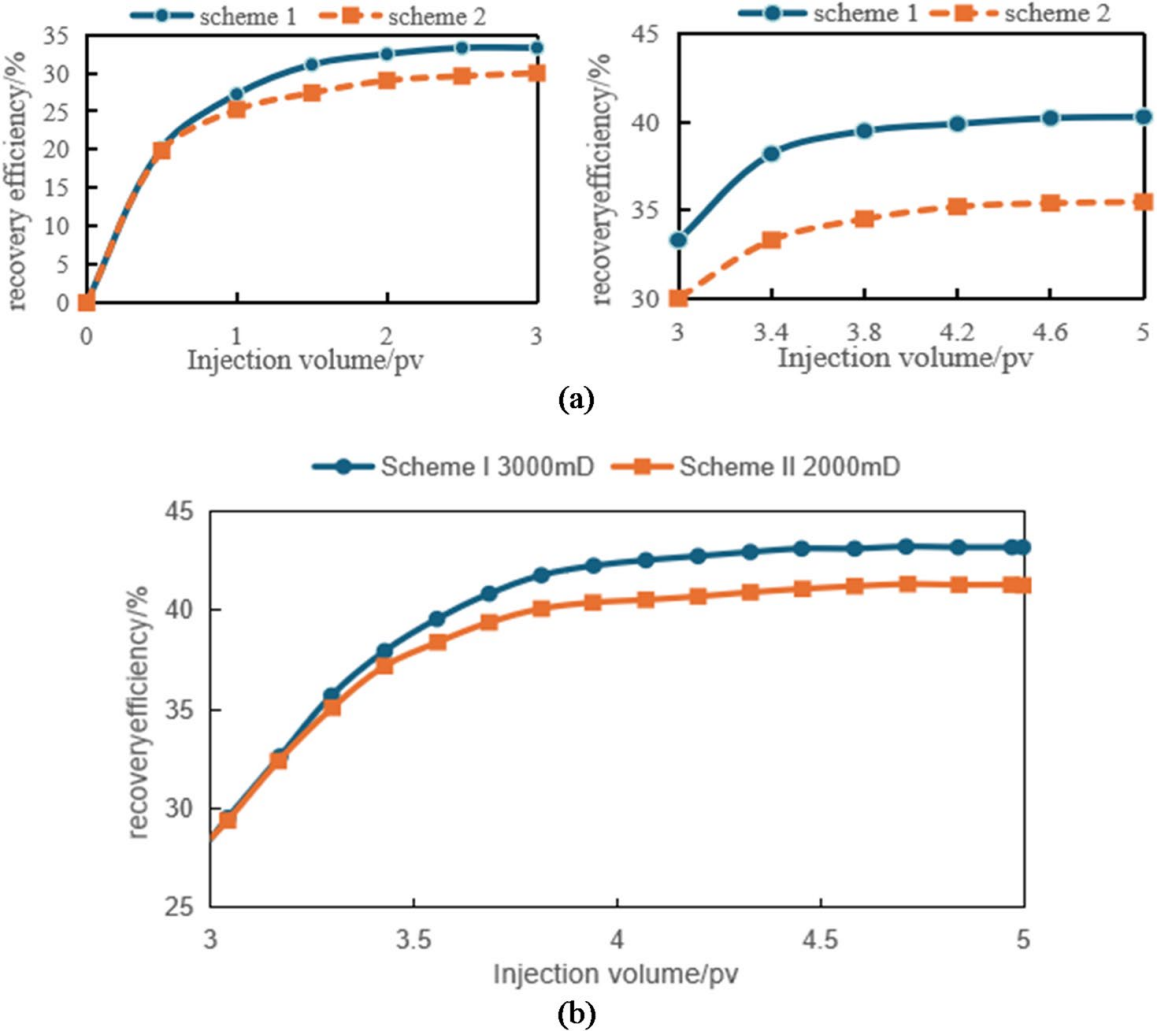
Comparison of recovery factors between development schemes 1 and 2. (a) Comparison of recovery factors between Scheme 1 and Scheme2, (b) Comparison of recovery factors in medium-permeability zones between Scheme I and Scheme II.

Comparative Analysis of Scheme 1 and Scheme 2: As demonstrated in the preceding discussion, shutting down the high-permeability section of horizontal wells during 3-5PV significantly enhances the overall reservoir recovery rate. For Scheme 1, the recovery rate in medium-permeability segments.

increased by 15% points, while that in low-permeability segments improved by 5.9% points. Scheme 2 showed a 12.9% point increase in medium-permeability segments, but only a 4% point improvement in low-permeability segments (Fig. 16a). This analysis demonstrates that Scheme 1 demonstrates stronger recovery enhancement effects across different permeability segments.

The trend is further illustrated by the graph of permeability variation in the medium-permeability zone. (Fig. 16b) demonstrates the dynamic evolution of recovery factor in the medium-permeability zone during the 3–5PV injection phase. It is evident that as injection volume increases, the medium-permeability zone becomes the primary contributor, with its recovery rate growth significantly outpacing that of the low-permeability zone. Throughout the injection period, Scheme 1 consistently outperforms Scheme 2 in recovery rate curves, exhibiting a faster upward trajectory. This indicates Scheme 1 demonstrates superior advantages in optimizing injection-production flowlines and suppressing inter-segment interference.

As shown in (Fig. 16a), the permeability gradient serves as a key factor influencing recovery efficiency. During the 0-3PV stage, Scheme I (permeability gradient 1.67/3/5) achieved 10.9% points higher overall recovery compared to Scheme II (permeability gradient 3.35/6.67/220.3). By the end of the experiment, this advantage expanded to 13.5% points, demonstrating that a lower permeability gradient enhances overall development effectiveness. Figure 24 clearly illustrates the recovery rate trends of both Schemes during the 0-3PV stage, with Scheme I’s curve consistently outperforming Scheme II, visually highlighting its development advantages.

**Fig. 17:**
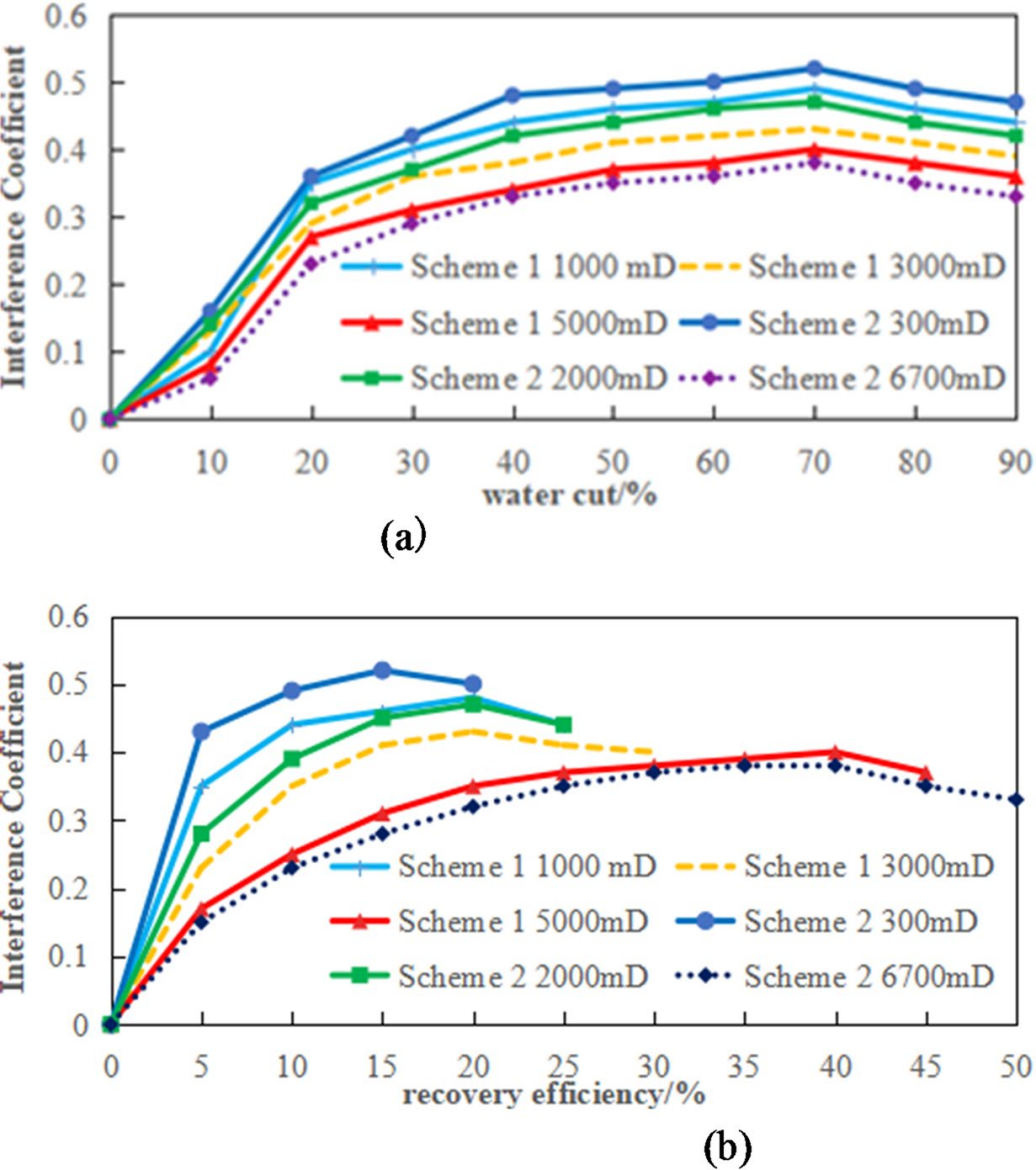
Schematic diagram of interference coefficient variation with production rate. (a) Schematic illustration of the 0-3PV interference coefficient variation with Water cut, (b) schematic diagram of 0-3PV interference coefficient variation with production rate.

Figure 17a and b demonstrate the intrinsic relationship between interference coefficients and oil recovery factor. The images reveal that the interference coefficients in the high-permeability, medium-permeability, and low-permeability segments of Scheme II are 0.022, 0.036, and 0.041 higher than those in Scheme I, respectively. This indicates that the greater the permeability gradient, the more severe the inter-segment interference becomes, with the low-permeability segments showing particularly significant interference.

The comprehensive experimental results demonstrate that Scheme 1, featuring a smaller permeability gradient compared to Scheme 2, achieves superior recovery factor through the closure of high-permeability segments. Analysis of the permeability-recovery curve reveals that the recovery enhancement primarily stems from the mid-permeability zone. This section exhibits improved fluid mobility and reduced inter-segment interference effects, resulting in significant recovery efficiency gains. Although the low-permeability zone also shows some recovery increase, its persistent exposure to substantial inter-segment interference limits the improvement, ultimately failing to meet the anticipated enhancement targets.

Further analysis of key factors affecting oil recovery factor reveals that permeability gradient and interference coefficient are primary influencing factors^5^. When the permeability gradient is significant, uneven fluid distribution between different permeability segments leads to fluid “short-circuiting” in high-permeability areas, thereby intensifying inter-segment interference effects and suppressing recovery efficiency in low-permeability segments. Optimizing injection-production flowlines to reduce inter-segment interference, particularly in low-permeability segments, is crucial for enhancing overall recovery factor.

### Verification of numerical and physical models and analysis of residual oil distribution

#### Verification of recovery curves from numerical and physical models

**Fig. 18:**
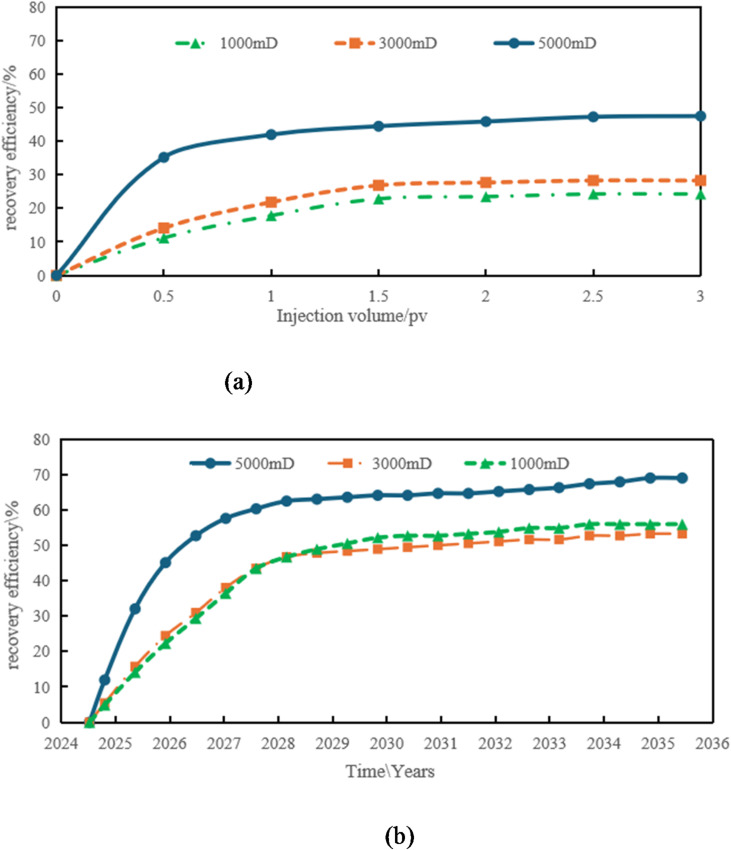
Comparison of production data between scheme 1 and scheme 2. (a) Time variation of recovery rate under numerical simulation, (b) variation of recovery rate over time under the physical model.

Figure 18a and b demonstrate the comparison of recovery curves between numerical simulations and physical experiments under different permeability conditions. The figures reveal strikingly similar trends in recovery rate variations between the two methods, with both showing consistent upward progression as the injection volume increases. For all three permeability levels (1000mD, 3000mD, 5000mD), the recovery curves exhibit a characteristic pattern: initial rapid growth followed by gradual deceleration before stabilizing.

In low-permeability reservoirs (1000 mD), the recovery rate shows a significant increase during the initial injection phase. However, as the injection volume continues to rise, the rate of recovery improvement gradually slows down and eventually stabilizes. This indicates that under low-permeability conditions, waterflooding has limited effectiveness. While the efficiency of waterflooding is relatively high in the early stage, it becomes incomplete in later stages, resulting in uneven distribution of remaining oil.

Reservoirs with medium permeability (3000 mD) and high permeability (5000 mD) exhibit similar trends, but their recovery rate increases at a slower pace. The rate of recovery growth becomes notably more gradual in high-permeability reservoirs, particularly when permeability is exceptionally high. This indicates that in high-permeability reservoirs, while the waterflooding front advances more uniformly, the accelerated water breakthrough and more evenly distributed remaining oil ultimately limit the further improvement of recovery factor.

The comparative analysis of numerical simulation data and physical experimental data reveals that both approaches demonstrate remarkably consistent trends in overall recovery rate variation. Both methods show a gradual increase in recovery rate with rising injection volume, and under identical permeability conditions, their data changes exhibit striking consistency. Whether operating at low, medium, or high permeability levels, the predicted results from numerical simulations and the actual measured values from physical experiments display similar curve characteristics. Particularly during the initial recovery rate increase phase, the two methods synchronize almost perfectly, indicating a high degree of compatibility in capturing the patterns of water drive processes and the distribution of remaining oil.

The alignment of this trend validates the reliability and accuracy of numerical simulations in capturing water drive efficiency during reservoir development. Through numerical simulations, we can predict water drive performance at different development stages of oil fields in advance, providing references for subsequent development decisions. Meanwhile, the results of physical experiments provide field validation for numerical simulations, demonstrating the effectiveness of the assumptions and models used in these simulations.

Therefore, the closeness between numerical simulation and physical experimental data not only enhances the reliability of simulation method, but also provides a scientific basis for the evaluation of water drive effect and the optimization of oil recovery rate in the process of oilfield development.

#### Effects of injection–production configuration on residual oil distribution

**Fig. 19:**
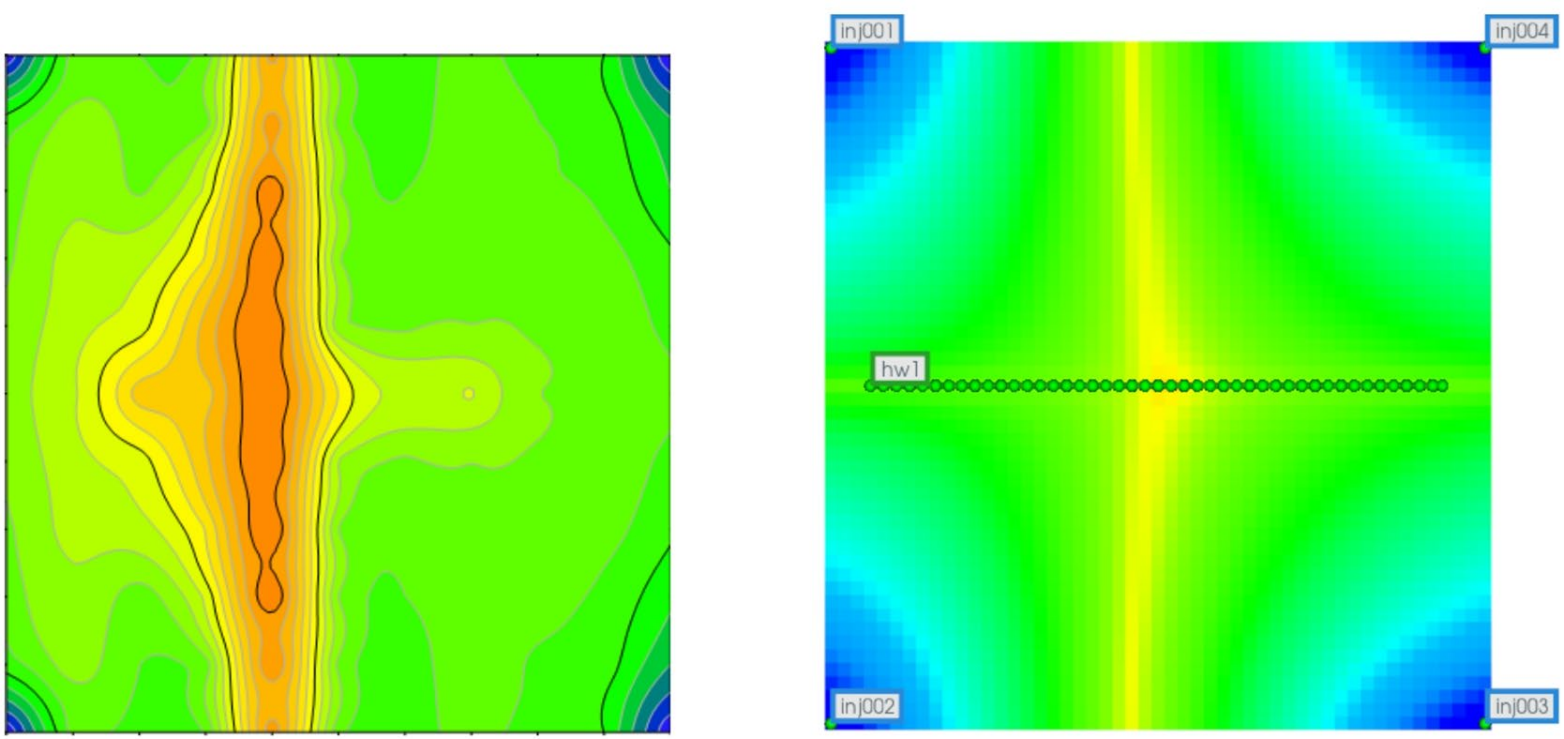
Comparison of remaining oil distribution between numerical simulation and 3D physical model for Scheme 1.

Based on the above validation analyses using numerical simulation and physical modeling, a high degree of agreement with small discrepancies was observed, providing a reliable basis for further investigation of residual oil distribution. As shown in the Fig. 19, after waterflooding, the spatial distribution of residual oil exhibits a pronounced segmented characteristic. Residual oil is mainly concentrated in the middle segment of the model, whereas the two end segments contain relatively less residual oil and show a more complete recovery. The middle segment exhibits the lowest recovery efficiency and represents the primary residual oil enrichment zone within the entire displacement system, indicating an evident insufficiency in sweep efficiency^17,18^.

**Fig. 20:**
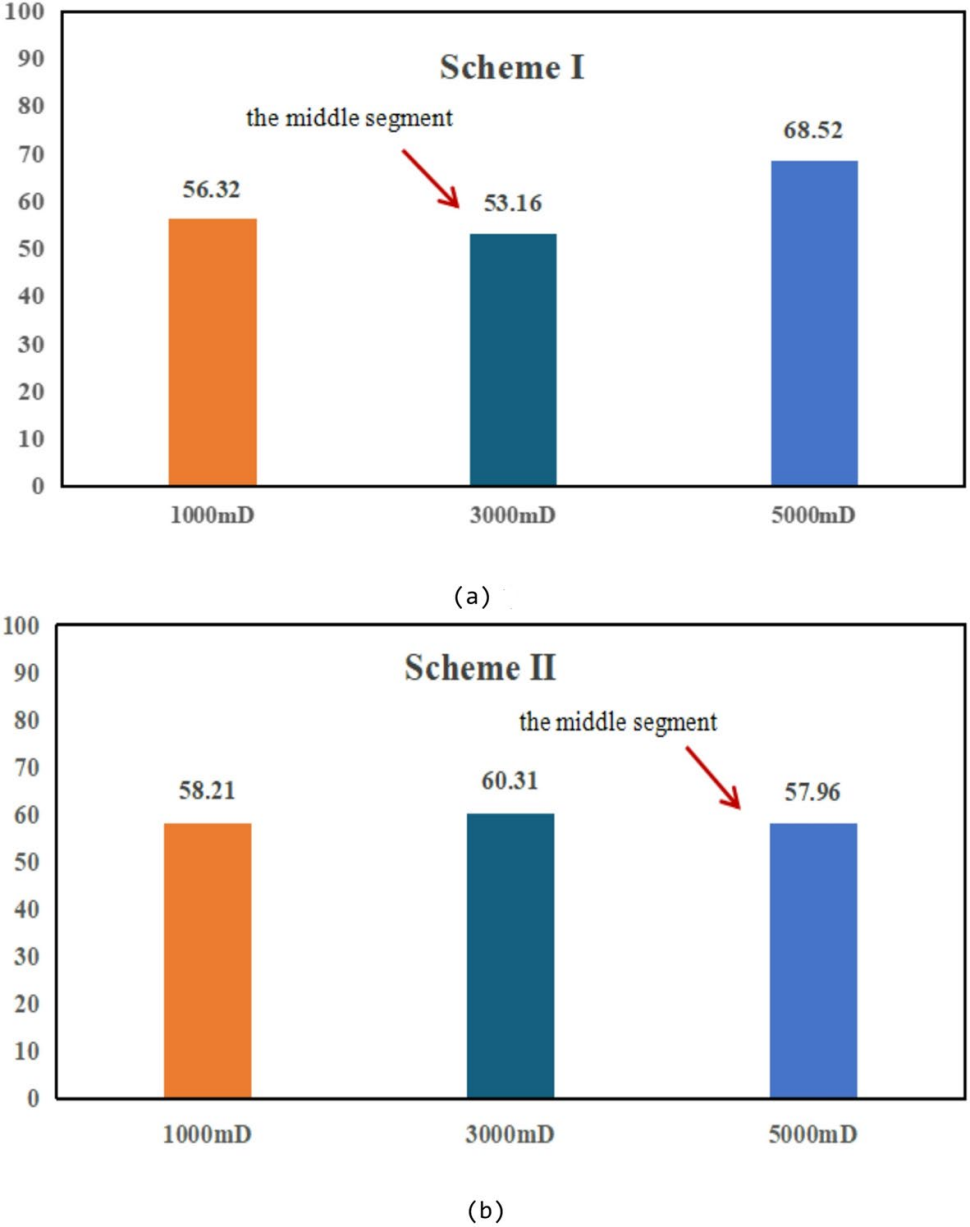

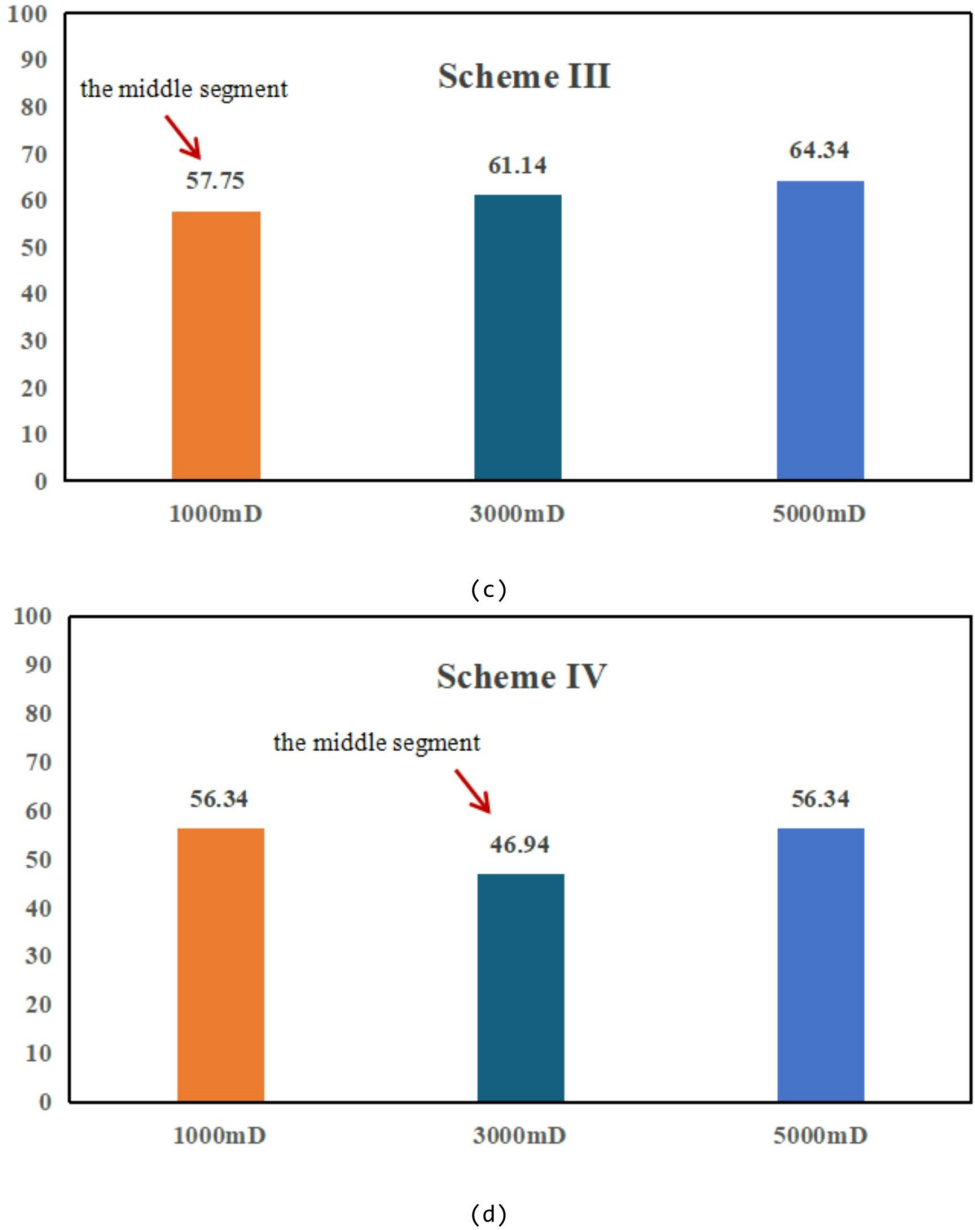
Comparison of middle-segment recovery efficiency under different permeability rhythm conditions. (a) Recovery efficiency of the middle segment under different permeability conditions, (Scheme I), (b) Middle-segment recovery efficiency under different permeability conditions for Scheme II, (c) Middle-segment recovery efficiency under different permeability conditions for Scheme III, (d) Middle-segment recovery efficiency under different permeability conditions for Scheme IV.

Based on the observations presented in Fig. 20, four sets of numerical simulation experiments were conducted. Scheme I corresponds to a positive permeability rhythm (1000 mD, 3000 mD, 5000 mD); Scheme II represents a reverse permeability rhythm (1000 mD, 5000 mD, 3000 mD); Scheme III is a composite permeability rhythm (3000 mD, 1000 mD, 5000 mD); and Scheme IV is a homogeneous model (1000 mD).

**Fig. 21:**
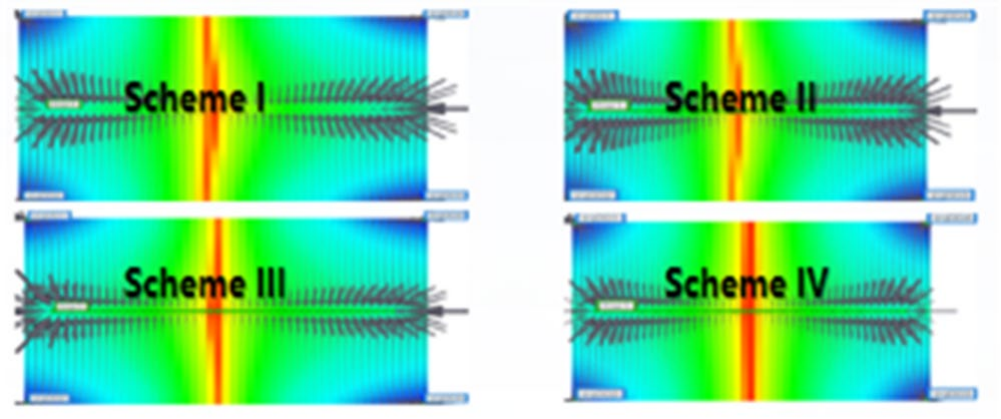
Oil saturation of different segments under different schemes.

By comparing the variations in recovery efficiency of the middle segment and the overall system under different schemes, it is observed that the recovery efficiency of the middle segment is significantly lower than that of the two end segments in all cases. Specifically, the middle-segment recovery efficiencies are 53.16% for Scheme I (Fig. 20a), 57.96% for Scheme II (Fig. 20b), and 57.75% for Scheme III(Fig. 20c), while the homogeneous model (Scheme IV) (Fig. 20d) exhibits the lowest middle-segment recovery efficiency of only 46.94%. Although the permeability rhythm distributions differ among the schemes, the middle segment consistently shows the lowest recovery efficiency, indicating that residual oil enrichment in this segment exhibits a strong stability (Fig. 21).

The differences in recovery efficiency among the schemes are mainly controlled by the effect of reservoir permeability heterogeneity on waterflood sweep efficiency. Under different permeability distributions, the flow paths and sweep extent of injected water vary significantly, thereby affecting displacement efficiency and recovery performance. Since no injection well is deployed in the middle segment, its displacement process relies entirely on the water injection from the two end segments. Consequently, the recovery efficiency of the middle segment is influenced not only by its intrinsic permeability but also directly constrained by the permeability contrast between the middle segment and the adjacent end segments. Under this injection–production configuration, inter-segment interference is primarily induced by water injection at the two ends, where injected water preferentially advances toward the end segments, resulting in insufficient displacement energy and limited sweep coverage in the middle segment. Owing to the lack of direct injection support, effective displacement channels are difficult to form in the middle segment, leading to long-term accumulation of residual oil in this region. Numerical simulation results further demonstrate that, even when the permeability rhythm distribution is adjusted, the middle segment remains the least efficiently recovered zone under the current injection–production conditions, and the residual oil enrichment pattern in this segment exhibits strong stability.

### In-depth investigation of the utilization pattern of horizontal well sections and its impact on production capacity

The preceding analysis confirmed the accuracy of the numerical model through comparative validation with physical model experiments. Building on this conclusion, we conducted in-depth quantitative analysis using the numerical model. This model provides an efficient and precise tool for predicting variations in recovery factor and inter-well interference effects under different operating conditions.

#### Pattern of horizontal well sectionalization and its impact on production capacity: permeability

Table 8 recovery factor and interference coefficients under different permeability gradient levels reveals significant differences in recovery factor and interference coefficients across five groups with varying permeability gradient differences. Permeability gradient differences decisively influence both inter-segment interference intensity and overall recovery factor in horizontal well sections. The recovery factor were 47.95%,47.13%,46.45%,43.92%, and 43.76%, with corresponding interference coefficients of 0.11,0.13,0.16,0.29, and 0.31. As permeability gradient differences increased, the fluid dominance channel effect in high-permeability segments intensified, significantly enhancing their mobilization efficiency. Conversely, low-permeability segments experienced continuous deterioration in mobilization capacity due to strong suppression from high-permeability sections, ultimately leading to decreased overall recovery factor and increased inter-segment interference coefficients. See Table 8 for recovery factor and interference coefficients under different permeability gradient differences.

**Table 8:** Recovery factor and interference coefficients under different permeability gradient levels.

Permeability contrast gap	Low-permeability segment	High-permeability segment	Recovery ratio	Interference factor
1.5	2400mD	3600mD	47.95%	0.11
2	2000mD	4000mD	47.13%	0.13
3	1500mD	4500mD	46.45%	0.16
4	1200mD	4800mD	43.92%	0.29
5	1000mD	5000mD	43.76%	0.31

Further analysis reveals a critical threshold (approximately 3): when the permeability gradient falls below 3, the decline in recovery rate and the increase in interference coefficient show relatively gentle trends. However, when the gradient exceeds 3, both exhibit sharp nonlinear growth characteristics. This phenomenon is visually demonstrated by the minimal remaining oil distribution observed at a permeability gradient of 3.

Accordingly, the engineering upper limit of permeability gradient difference is determined as 3, which provides a key design basis for the balanced utilization and development scheme optimization of this type of reservoir.

#### Pattern of horizontal well sectional operation and its impact on production capacity: pressure difference between injection and production

Numerical simulation results demonstrate that the injection-production pressure differential gradient significantly influences interference intensity and development effectiveness between horizontal well sections. As the gradient increases, the driving advantage in high-pressure differential segments induces fluid migration, severely limiting the effective water injection coverage efficiency in low-pressure differential segments. This prevents effective oil displacement in these segments, ultimately resulting in reduced overall recovery factor. Table data shows that increasing the injection-production pressure differential gradient leads to continuous decline in recovery factor and steady rise in interference coefficients (Table 9).

**Table 9:** Recovery and interference coefficient under different injection-production pressure differences.

Injection-production pressure difference	Low production pressure gradient zone	High injection- production pressure difference section	Recovery ratio	Interference factor
1.5	1.6	2.4	59.04%	0.25
2	1.33	2.67	58.78%	0.27
2.5	1.14	2.86	56.55%	0.39
3	1	3	56.17%	0.42
3.5	0.89	3.11	55.97%	0.44

Further analysis reveals a clear nonlinear relationship between the injection-production pressure differential gradient and development indicators: when the gradient is less than 2, the decline in recovery rate and the increase in interference coefficient are relatively gentle; however, when the gradient exceeds 2, the inter-segment interference effect is dramatically amplified, and the recovery rate decreases rapidly.

Based on this mutation feature, the upper limit of engineering control of injection-production pressure difference gradient is determined as 2, which provides a key basis for the design and optimization of reasonable production pressure difference of this type of reservoir.

#### Pattern of horizontal well sectional activation and its impact on production capacity: water saturation contrast

Numerical simulation results show that the increase of water content gradient significantly aggravates the interference effect between horizontal well sections. The fundamental reason is the systematic increase of the inter-segment interference coefficient (Table 10).

**Table 10:** Recovery and interference coefficient at different water content levels.

Water saturation contrast	Low water content section	High water content section	Recovery ratio	Interference factor
1.05	0.83	0.87	43.39%	0.11
1.10	0.81	0.89	42.82%	0.15
1.15	0.79	0.91	39.84%	0.28
1.2	0.77	0.93	39.37%	0.33
1.25	0.76	0.94	39.12%	0.35

Further research found that there is a clear critical feature between the water saturation contrast and the development index: when the gradient is less than 1.1, the decline of the recovery rate and the rise of the interference coefficient are relatively limited; but once the gradient is more than 1.1, both of them show a sharp deterioration trend.

Based on this nonlinear turning feature, the upper limit of engineering control of moisture gradient is determined as 1.1.

#### Pattern of horizontal well sectional development and its impact on production capacity: thickness variation across segments

Numerical simulation results demonstrate that reservoir thickness gradients primarily influence recovery factor through macroscopic water injection efficiency. As thickness gradients increase, gravity segregation and permeability resistance differences enable injected water to preferentially penetrate thick reservoir segments with rapid wave propagation. This process significantly reduces water injection effectiveness in thin layers, where crude oil remains trapped and difficult to displace, ultimately leading to overall recovery rate decline (Table 11).

**Table 11:** Reservoir recovery and interference coefficients under different thickness gradients.

Thickness difference	Thin	Thick	Recovery ratio	Interference factor
1.5	24	36	46.4%	0.381
2	20	40	46.13%	0.382
2.5	17.14	42.86	45.85%	0.386
3	15	45	45.13%	0.387
3.5	13.33	46.67	45%	0.387

Further analysis revealed a distinct nonlinear relationship between thickness gradient and development effectiveness: when the gradient is below 2, the decline in recovery rate and the increase in interference coefficient show relatively gentle trends. However, when the gradient exceeds 2, the permeation difference between thick and thin layers becomes dramatically pronounced, resulting in a sharp drop in recovery rate and a simultaneous surge in interference coefficient .

Based on this critical feature, the upper limit of engineering control of thickness gradient is determined as 2.

### Orthogonal analysis

#### Orthogonal analysis approach

To systematically quantify the impacts of permeability, water cut gradient, injection-production pressure difference, and formation thickness on waterflooding development effectiveness, this study employed orthogonal analysis through 20 experimental schemes covering multiple factors and levels. After conducting dynamic displacement simulations for each scheme using numerical modeling, orthogonal analysis was performed to precisely quantify the relative influence of each factor. This approach enabled reliable identification of key controlling factors that constrain waterflooding efficiency^19,20^.

Through orthogonal analysis, the results show that: Based on this hierarchy, permeability and thickness differences significantly affect waterflooding efficiency. Specifically, high-permeability segments tend to form dominant flow channels, causing premature breakthrough of water flow and substantially reducing sweep efficiency. In low-permeability segments, a notable synergistic effect exists between injection-production pressure difference and water cut gradient. Their combined effect intensifies displacement heterogeneity, severely limiting the effective utilization of remaining oil. Through simulation, the critical thresholds for water cut gradient, permeability, injection-production pressure difference, and reservoir thickness were identified.

#### Experience equation construction

To address the inter-segment interference commonly observed during multi-segment cooperative development of reservoirs at the ultra-high water-cut stage, a quantitative characterization method for inter-segment interference was established based on numerical simulation results. First, reservoir numerical simulation models were employed to calculate the recovery performance under separate production and commingled production conditions for different levels of permeability contrast, water saturation contrast, thickness contrast, and injection–production pressure contrast. On this basis, an interference coefficient, $\beta$, was introduced to quantitatively describe the inter-segment interference effect, which is defined as the normalized difference between the recovery under separate production and that under commingled production.

11 $$\beta =\frac{{{q_{{\mathrm{sep}}}} - {{\mathrm{q}}_{{\mathrm{com}}}}}}{{{{\mathrm{q}}_{{\mathrm{sep}}}}}}$$

where ${q_{{\mathrm{sep}}}}$and ${q_{{\mathrm{com}}}}$denote the recovery under separate production and commingled production conditions, respectively. After obtaining the interference coefficients corresponding to different levels of controlling factor contrasts, statistical regression analysis was performed on the simulation results using SPSS software. Based on previous studies on inter-segment interference and reservoir heterogeneity characterization, permeability contrast, water saturation contrast, thickness contrast, and injection–production pressure contrast were selected as the dominant independent variables. A nonlinear regression method was then applied to establish an empirical relationship between the interference coefficient and the controlling factors. The resulting interference formula preserves the essential physical characteristics of inter-segment interference, particularly its sensitivity to reservoir heterogeneity and injection–production conditions, while the model parameters were calibrated using numerical simulation results. Therefore, the proposed formula can be regarded as a semi-empirical, semi-analytical model derived from numerical simulation data.

$$\beta =0.12+0.28\lambda _{k}^{{0.75}}+0.35{\left( {{\lambda _{{f_{\mathrm{w}}}}} - 1} \right)^{3.2}}$$

12 $$+ 0.12\ln \lambda _{{\Delta {\mathrm{p}}}} + 0.38\left( {1 - {\mathrm{e}}^{{ - 0.5\lambda _{{\mathrm{h}}} }} } \right) - 0.02\lambda _{{\mathrm{k}}} \ln \left( {\lambda _{{\Delta {\mathrm{p}}}} } \right){\mathrm{~}}$$

where λ_K_, λ_sw_, λ_h_, and λ_ΔP_ denote the permeability contrast, water saturation contrast, thickness contrast, and injection–production pressure difference, respectively, and β represents the interference coefficient.

**Fig. 22:**
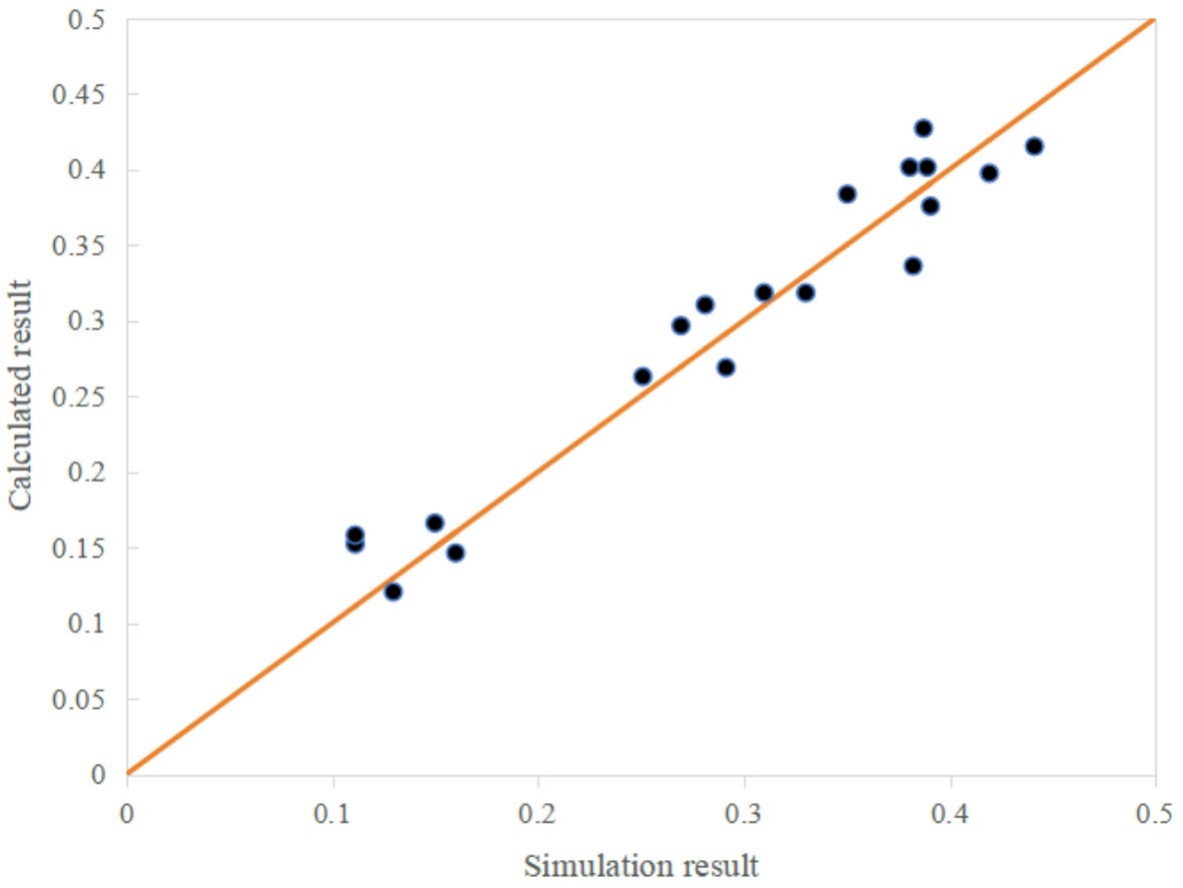
Comparison between calculated results and simulation results.

To verify the rationality and applicability of the proposed interference formula, the calculated results obtained from the formula were further compared with numerical simulation results. The results indicate that the calculated values from the interference formula show good agreement with the simulation results (Fig. 22), with data points generally distributed near the ideal consistency line and no evident systematic deviation observed. This demonstrates that the proposed formula can accurately capture the variation of inter-segment interference intensity under different operating conditions, exhibiting high fitting accuracy and good potential for engineering application.

## Conclusions and outlook

This study conducted an in-depth analysis of how the “Horizontal - Vertical” interference pattern affects remaining oil enrichment patterns in Bohai Oilfield during the ultra-high water cut period. By combining physical and numerical simulations, the research revealed the significant impact of inter-segment Interference between injection and production segments on waterflooding processes. The findings led to the following conclusions:

1. The controlling factor analysis indicates that when the permeability ratio exceeds 3, injected water preferentially flows into high-permeability zones, leading to reduced sweep efficiency and significant recovery differences. Keeping the permeability contrast below 3 helps improve displacement uniformity and overall recovery. The water saturation contrast has a strong influence on the distribution of remaining oil; when controlled within 1.1, it stabilizes the waterflood front, suppresses ineffective circulation, and facilitates the mobilization of remaining oil. A pressure differential contrast greater than 2 increases inter-segment interference, whereas maintaining it below 2 enhances waterflood uniformity. The impact of thickness contrast on recovery is relatively weak; when it exceeds 2, recovery declines rapidly, and inter-segment interference intensifies, thus setting an upper limit of 2 for thickness contrast.
2. Based on four numerical simulation scenarios, the differences in recovery among the schemes are primarily controlled by the effect of reservoir permeability heterogeneity on waterflood sweep efficiency. In the middle segment, which lacks a dedicated injection well, displacement relies entirely on water injected from the two end segments; thus, recovery is influenced not only by the segment’s intrinsic permeability but also directly constrained by the permeability of the adjacent end segments. Inter-segment interference is mainly induced by end-segment injection, resulting in persistent accumulation of residual oil in the middle segment. The results further indicate that Scheme 3, with a segmental permeability configuration of 3000 mD-1000 mD-5000 mD, achieves the highest overall recovery, demonstrating that an appropriate arrangement of segmental permeability can optimize sweep efficiency and enhance overall recovery.
3. To quantitatively assess interference effects, a calculation formula and evaluation.system for interference coefficients were established. This model can predict interference effects under different water-bearing stages based on parameters such as oilfield development phase, permeability, and injection-production flow rates. It provides an effective tool for predicting water drive efficiency and remaining oil distribution, offering scientific support for long-term oilfield development decisions.
4. While this study has established fundamental principles of interference effects within injection-production segments, several factors remain underexplored. For instance, reservoir fluid heterogeneity and instability may further compromise waterflood efficiency, suggesting future research should prioritize investigating fluid dynamics’ impact on water displacement processes. Moreover, as oil fields progress into mid-to-late development stages, remaining oil distribution may be influenced by additional factors such as inter-well interference and inter-layer connectivity. Therefore, it is crucial to conduct further experiments and simulations to optimize waterflood effectiveness at different development phases^21–23^.

## Data Availability

To access the measured experimental data, one can contact Ma Kuiqian by contacting email of m25515461@163.com.
